# Zhen-Wu-Tang ameliorates uremic cardiomyopathy via targeting the kidney–heart inflammatory axis and suppressing CCL2/CCR2-mediated macrophage activation

**DOI:** 10.1186/s13020-026-01376-2

**Published:** 2026-03-18

**Authors:** Yu Xu, Jing Cai, Yuan-Ming Fan, Lian-Wen Qi, Lei Zhang

**Affiliations:** 1 Pukou Hospital of Chinese Medicine, China Pharmaceutical University, Nanjing, 211198 China; 2 School of Traditional Chinese Pharmacy, China Pharmaceutical University, Nanjing, 211198 China; 3 Clinical Metabolomics Center, China Pharmaceutical University, Nanjing, 211198 China

**Keywords:** Zhen-Wu-Tang, Uremic cardiomyopathy, Cardiorenal syndrome, Inflammation, Macrophages

## Abstract

**Background:**

Zhen-Wu-Tang (ZWT), a classic herbal formula from Treatise on Febrile and Miscellaneous Diseases, is commonly used for heart and kidney-related diseases. Despite its widespread application, research on the active components of ZWT and their mechanisms in heart–kidney cross-organ regulation remains underexplored.

**Aim of the study:**

This study aimed to elucidate the therapeutic mechanisms of ZWT in uremic cardiomyopathy (UC) focusing on its modulation of the heart–kidney inflammatory axis.

**Materials and methods:**

A UC model was established via 5/6 nephrectomy in mice, followed by 8 weeks of ZWT treatment. Functional assessments included serum creatinine, blood urea nitrogen, cardiac ejection fraction, and left ventricular metrics. Proteomic analysis using Olink technology exerts its therapeutic effects by suppressing systemic inflammation. UHPLC-Q/TOF–MS were employed to identify prototype components and blood-entering components in ZWT. Cellular experiments using a three-step co-culture system were conducted to evaluate the regulatory effects of ZWT active components on HK-2 and AC16 cells and to explore their underlying molecular mechanisms.

**Results:**

ZWT significantly improved renal and cardiac functions. Proteomics revealed ZWT suppressed pro-inflammatory cytokines TNFα, IL-6, IL-1β and chemokines. The bioactive constituents of ZWT, including benzoylaconine, paeoniflorin, and atractylenolide III, inhibited NF-κB activation, thereby reducing CCL2 synthesis and subsequent macrophage recruitment via the CCR2 axis. This attenuated systemic inflammation and cardiomyocyte injury.

**Conclusions:**

ZWT exerts therapeutic effects on UC by targeting the kidney-heart inflammatory axis and suppressing CCL2/CCR2-mediated macrophage activation. This study provides new insights into the molecular mechanisms underlying ZWT's efficacy in treating heart–kidney disorders.

**Supplementary Information:**

The online version contains supplementary material available at 10.1186/s13020-026-01376-2.

## Highlights

- This study reveals the dual cardiorenal protective effects of ZWT in uremic cardiomyopathy.
- A total of 45 major compounds and 34 blood-entering components are identified in ZWT using UHPLC-Q/TOF–MS analysis.
- The cardiorenal protective effects of ZWT are mediated through targeting the kidney-heart inflammatory axis and suppressing CCL2/CCR2-mediated macrophage activation.

**Supplementary Information:**

The online version contains supplementary material available at 10.1186/s13020-026-01376-2.

## Introduction

Uremic Cardiomyopathy (UC) is a common and irreversible cardiovascular complication in chronic kidney disease (CKD) patients, particularly those with End-Stage Kidney Disease (ESKD) undergoing dialysis [1]. Characterised by left ventricular hypertrophy, dilation, increased ventricular stiffness and diastolic dysfunction, UC often presents with preserved left ventricular ejection fraction [2]. It affects to 50% of CKD patients, significantly increases the risk of cardiovascular events and mortality [3]. The complexity of kidney–heart-vasculature crosstalk presents significant challenges in defining, researching, and identifying targeted and effective pharmacological treatments, due to the intricate interplay of multifaceted pathophysiological processes.

Multiple factors contribute to UC development, including sympathetic nervous system, over-activation of the renin–angiotensin–aldosterone system, endothelial injury, inflammation, oxidative stress, circulating uremic toxins, and renal anemia [4]. Among these, inflammation plays a central role in both acute and chronic cardiorenal dysfunction, as evidenced by elevated levels of circulating inflammatory mediators [5]. Chronic activation of pro-inflammatory molecules in the damaged kidneys leads to the recruitment of immune cells and increased production of cytokines and chemokines such as C-reactive protein (CRP) and tumor necrosis factor-α (TNFα) [6, 7]. In several animal models, TNFα has been shown to mediate progressive left ventricular (LV) dilation, dysfunction, and increased cardiac mortality. Additionally, interleukin-1β (IL-1β) contributes to adverse ventricular remodeling in chronic heart failure (CHF) by promoting cardiac apoptosis and extracellular matrix metabolism [8]. However, the specific biological sources and the underlying mechanisms driving this persistent inflammatory response remain incompletely understood, warranting further investigation.

Traditional Chinese Medicine (TCM) offers unique advantages in managing chronic diseases due to its holistic approach. Zhen-Wu-Tang (ZWT) derived from Treatise on Febrile and Miscellaneous Diseases by Zhang Zhongjing, an outstanding physician in the Han Dynasty [9, 10]. It is consists of Poria cocos, Paeoniae Radix Alba, Aconiti Lateralis Radix Praeparata, Zingiberis Rhizoma, Atractylodis Macrocephalae Rhizoma. Traditionally used to enhance renal function and regulate fluid metabolism, recent studies have highlighted its protective mechanisms in various kidney diseases [11]. Some studies showed that ZWT could induce mitophagy to protect mitochondrial function in chronic glomerulonephritis via the PI3K/AKT/mTOR and AMPK pathways [12]. It ameliorates lupus nephritis by reducing renal tissue-resident memory CD8 + T cells through IL-15/STAT3 suppression and attenuates Adriamycin-induced nephropathy by regulating AQP2 and miR-92b [13, 14]. Additionally, it can protect against IgA nephropathy and membranous nephropathy in rats by regulating exosomes to inhibit the NF-κB/NLRP3 pathway [15].

ZWT has been shown to improve cardiac function in murine models of isoproterenol-induced myocardial injury. This cardioprotection is achieved by attenuating myocardial fibrosis, suppressing TLR4/NF-κB pathways, and modulating the cardiac inflammatory microenvironment [16]. ZWT upregulates miR-451 expression in renal tissues, inhibits the TLR4/NF-κB/HIF-1α signaling loop, and attenuates renal fibrosis in rat models of type 2 cardiorenal syndrome induced by renal artery ligation and 3/4 nephrectomy [17]. However, existing studies predominantly focus on the individual effects of ZWT on either cardiac or renal function, as well as the associated molecular mechanisms. In contrast, its specific role in modulating the heart–kidney axis and the underlying mechanistic pathways remain poorly understood, highlighting the need for further investigation.

In this study, we investigated the dual cardiorenal protective mechanisms of ZWT. Using a UC mouse model, we found that ZWT significantly improved cardiac and renal dysfunction induced by 5/6 nephrectomy. We analyzed the chemical components of ZWT and identified the constituents absorbed into the bloodstream in rats using ultra-high-performance liquid chromatography coupled with quadrupole time-of-flight mass spectrometry (UPLC-Q-TOF–MS). Further, using plasma proteomics and co-culture systems involving renal tubular epithelial cells, macrophages, and cardiomyocytes, we found that active components of ZWT suppressed TNFα-induced chemokine release in renal tubular epithelial cells and mitigated CCR2 + macrophage recruitment. These effects led to reduced levels of inflammatory cytokines in the blood and ultimately alleviated cardiomyocyte hypertrophy. This study elucidates the regulatory mechanisms of ZWT on the heart–kidney axis, offering potential therapeutic insights for treating UC.

## Results

### ZWT ameliorated renal and cardiac dysfunction in mice with uremic cardiomyopathy

**Fig. 1:**
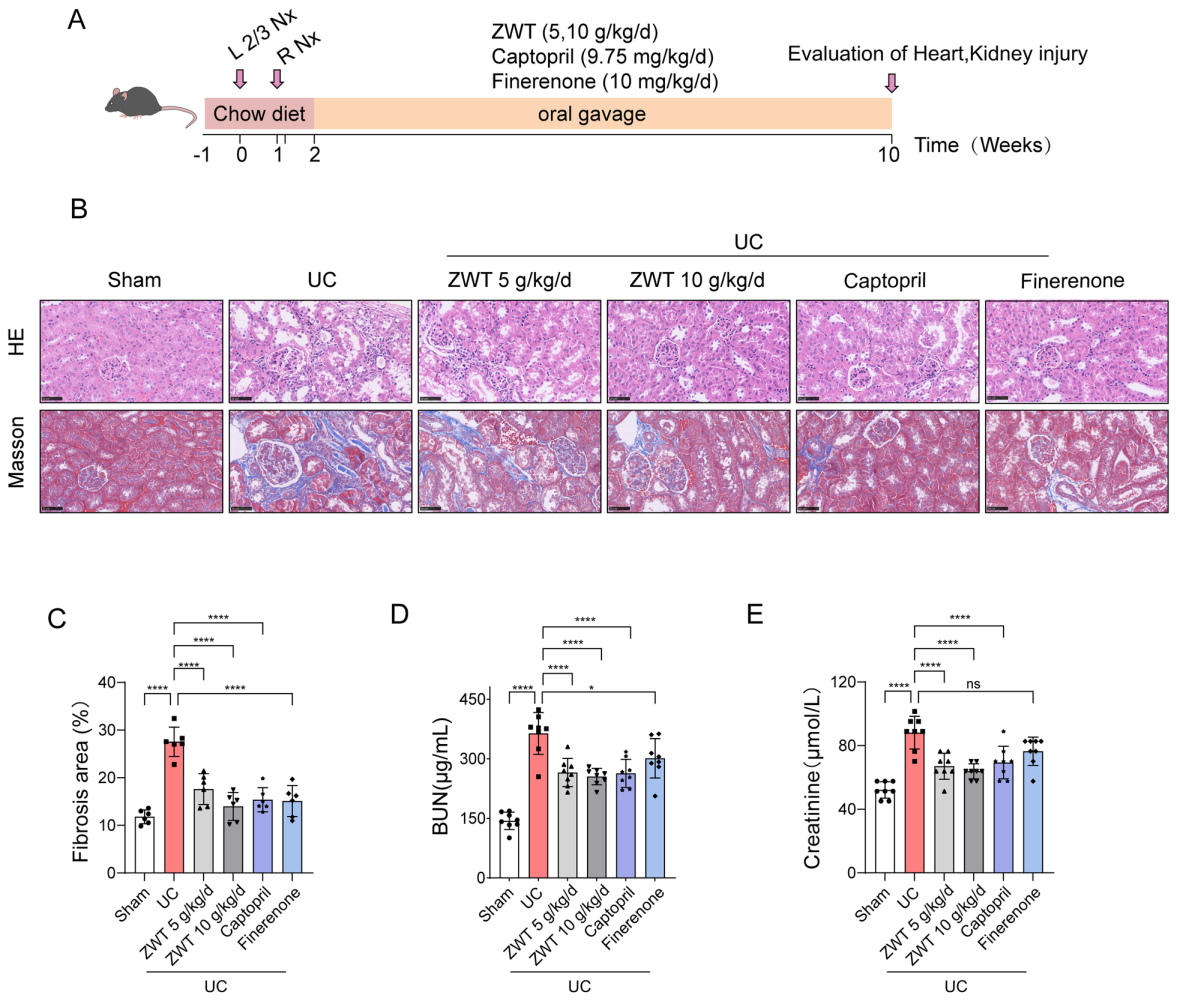
ZWT effectively alleviated uremic renal dysfunction in mice. ZWT Effectively Alleviated Uremic Renal Dysfunction in Mice. **A** Schematic of UC model establishment and ZWT treatment. Mice were administered ZWT at doses of 5 g/kg/d and 10 g/kg/d, Captopril (9.75 mg/kg/d), or Finerenone (10 mg/kg/d) for 8 weeks, following 5/6 nephrectomy. The sham group only underwent laparotomy and suturing, without kidney tissue resection. **B** Hematoxylin–eosin (HE) and Masson staining of kidney sections, n = 6 mouse kidneys per group, scale bar = 50 μm. **C** Quantification of the fibrotic area in Masson staining. **D** Blood urea nitrogen (BUN) levels and (**E**) Creatinine levels in blood were measured by ELISA, n = 8 mice per group. Data in **C–E** were presented as mean ± SD, data were analyzed using one-way ANOVA with Tukey's test, with *p* ≤ 0.05 considered statistically significant. *p ≤ 0.05, **p < 0.01, ***p < 0.001, ****p < 0.0001

A UC model was established in mice by 5/6 nephrectomy surgery. Following the surgery, the mice were administration of ZWT at doses of 5 g/kg/d and 10 g/kg/d for 8 weeks based on established effective and safe ranges reported in previous studies. [13, 16, 18], Captopril (9.75 mg/kg/d) [17] and Finerenone (10 mg/kg/d) [19] served as positive control (Fig. 1A). Morphologically, ZWT treatment effectively attenuated tubular dilatation and renal fibrosis (Fig. 1B, C), which was further supported by the downregulation of the injury marker *Kim1* and fibrosis-related genes including *Col1a1* and *Col3a1* (Fig. S1A-C), Consistent with the histological improvement, the markedly elevated serum BUN and creatinine levels in uremic mice were dose-dependently reduced by ZWT (Fig. 1D, E). The renal protection of ZWT was also associated with the suppression of inflammation. ZWT treatment markedly reduced F4/80 + inflammatory cell infiltration (Fig. S1D, E) and decreased the expression of pro-inflammatory cytokines such as *Tnfα* and *IL-1β* (Fig. S1F, G). Consistently, ZWT downregulated M1 macrophage markers including *iNOS* and *CD86* (Fig. S1H, I), suggesting an attenuation of the pro-inflammatory microenvironment. Furthermore, ZWT lowered malondialdehyde (MDA) levels (Fig. S1J) and reduced TUNEL-positive cell numbers (Fig. S1K, L). This anti-apoptotic effect was further confirmed by the decreased expression of Bcl2, alongside increases in Bax and Cleaved-Caspase3 levels (Fig. S1M-P). Collectively, these findings demonstrated that ZWT effectively alleviated renal injury, inflammation, and apoptosis in uremic mice.

**Fig. 2:**
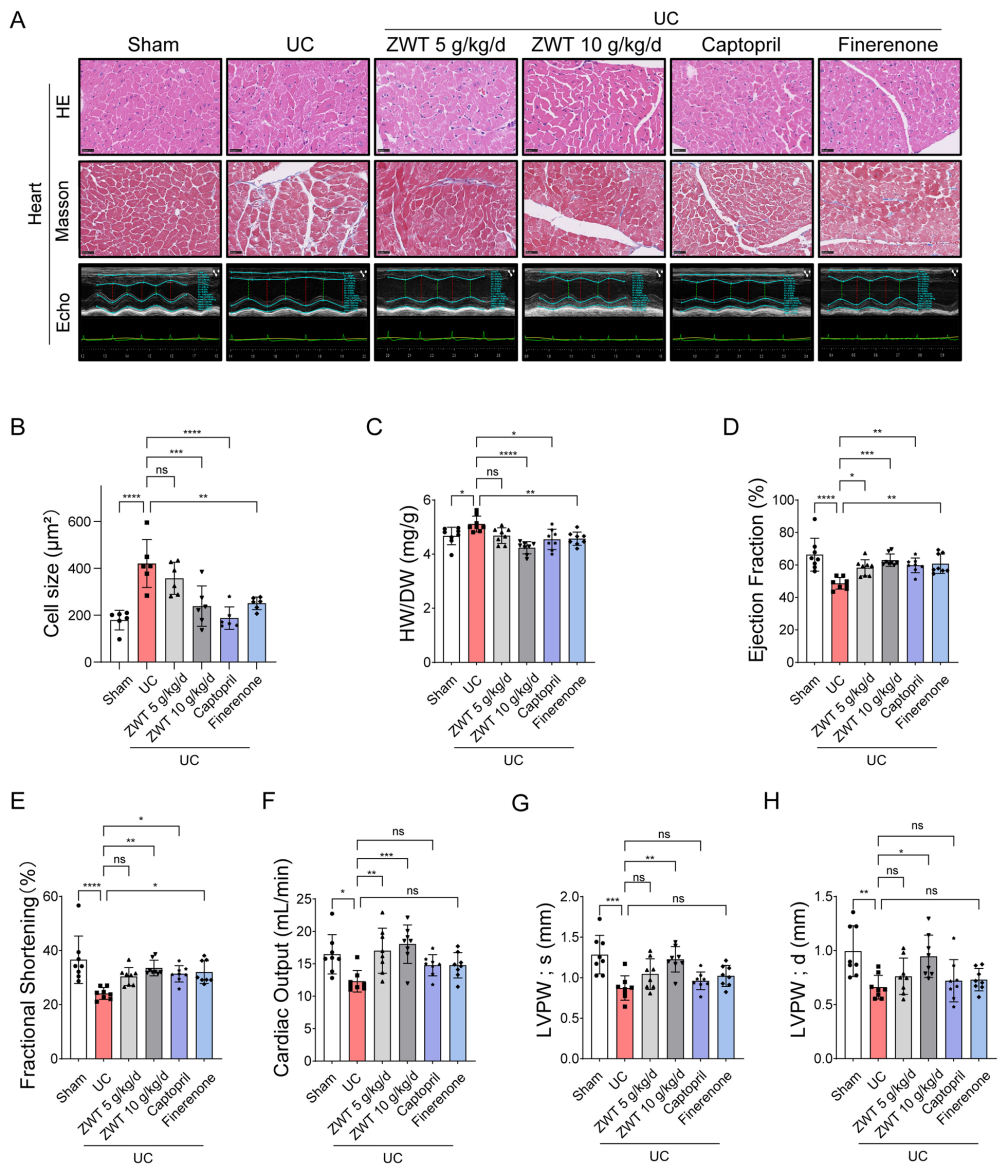
ZWT Improved UC in Mice. **A** Representative images of heart sections stained with HE, Masson’s trichrome, n = 6 mouse hearts per group, M-mode echocardiography from the mice 8 weeks after 5/6 nephrectomy with or without ZWT treatment, n = 8–10 mice per group, scale bar = 50 μm. **B** Quantification of cardiomyocyte size based on HE staining, n = 6 samples per group. **C–H** Statistical analysis of heart weight/body weight (HW/BW), ejection fraction (EF), fractional shortening (FS), cardiac output, left ventricular posterior wall thickness at end-systole (LVPW; s), and left ventricular posterior wall in at end-diastole (LVPW; d), n = 8 mice per group. Data in **B–H** were presented as mean ± SD, data were analyzed using one-way ANOVA with Tukey's test, with *p* ≤ 0.05 considered statistically significant. *p ≤ 0.05, **p < 0.01, ***p < 0.001, ****p < 0.0001

In the heart, HE and Masson staining demonstrated that ZWT significantly reduced uremia-induced cardiomyocyte hypertrophy (Fig. 2A, B). Consistently, heart weight which was elevated in uremic mice, was markedly reduced following treatment (Fig. 2C). Echocardiographic analysis revealed that ZWT reduced cardiac dilation and improved cardiac function, as evidenced by enhanced ejection fraction (Fig. 2D), fractional shortening (Fig. 2E), cardiac output (Fig. 2F), left ventricular posterior wall in systole (Fig. 2G), and left ventricular posterior wall in diastole (Fig. 2H). In addition, ZWT showed slightly better effect than positive control, especially, for cardiac output, LVPW; s and LVPW; d (Fig. 2F–H). Notably, ZWT also significantly attenuated uremia-induced atrial natriuretic peptide (*Nppa)* and B-type natriuretic peptide (*Nppb)* expression (Fig. S2A, B). These results suggest that ZWT improves cardiac function and reduces hypertrophy in UC. Furthermore, histological analysis of hepatic tissue revealed normal cellular architecture and tissue integrity, with no observable toxic effects of ZWT at either dosage (Fig. S3A). ALT and AST levels remained within normal ranges in all treatment groups (Fig. S3B, C), confirming the absence of hepatotoxicity.

### ZWT attenuated circulating inflammatory cytokines and chemokines

**Fig. 3:**
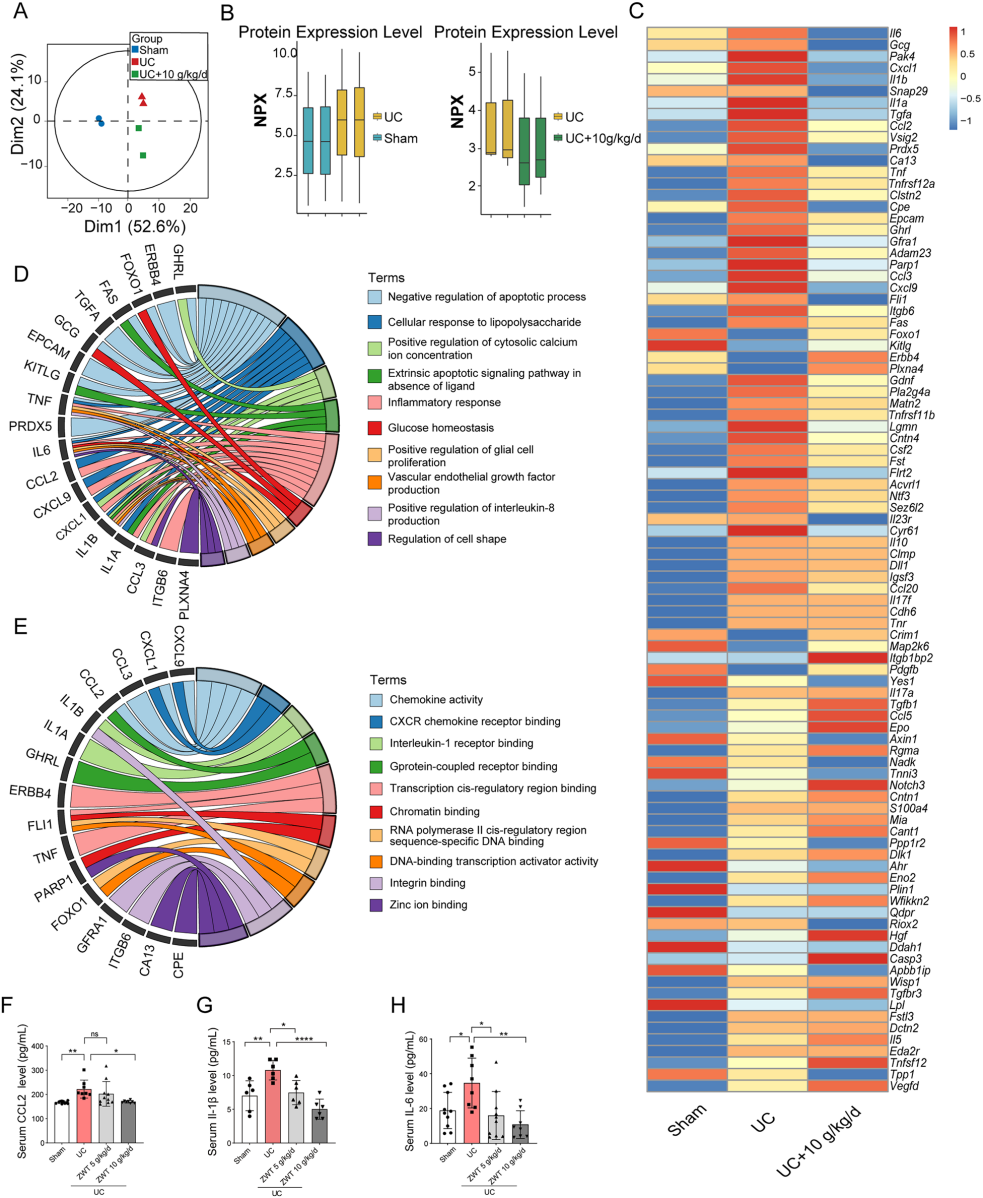
ZWT Attenuates Circulating Inflammatory Cytokines and Chemokines. ZWT Attenuates Circulating Inflammatory Cytokines and Chemokines. **A** Principal Component Analysis (PCA) of plasma secreted proteomics data comparing the sham group (blue), the UCM group (red), and the UCM with ZWT group (green), n = 2 samples per group. **B** Box plots showing expression values of samples in each group, n = 2 samples per group. **C** A heatmap illustrating the variations of 92 secreted proteins in mouse blood across the three groups. The data were presented as mean value of each group, n = 2 samples per group. **D–E** Gene Ontology (GO) enrichment analysis of biological processes (BP) and molecular functions (MF) for the top 30 proteins with the most significant reversed trends after ZWT treatment. **F** Circulating CCL2 levels were detected by ELISA, n = 8–10. Serum IL-1β levels quantified by ELISA, n = 6 mice per group. **H** Systemic IL-6 levels were measured by ELISA, n = 8–10 mice per group. Boxplots of B data were displayed the median as the center line, with the box limits representing the upper and lower quartiles. Data in **F–H** were presented as mean ± SD, data were analyzed using one-way ANOVA with Tukey's test, with *p* ≤ 0.05 considered statistically significant. *p ≤ 0.05, **p < 0.01, ***p < 0.001, ****p < 0.0001

To explore the dual cardiorenal protective mechanisms of ZWT, we employed the Olink proteomics analysis utilizing Proximity Extension Assay (PEA) technology. Principal Component Analysis (PCA) revealed distinct plasma protein profiles among the sham, UC, and ZWT treatment groups (Fig. 3A). Notably, ZWT broadly reversed 5/6 nephrectomy-induced alterations in circulating protein levels (Fig. 3B). Heatmap visualization further illustrated cytokine variations across the three groups, with specific cytokines including Interleukin-6 (IL-6), glucagon (GCG), C-X-C motif chemokine ligand 1 (CXCL1), interleukin 1 beta (IL-1β), transforming growth factor alpha (TGF-α), and C–C motif chemokine ligand 2 (CCL2), exhibiting a trend toward normalization following ZWT administration (Fig. 3C). The Gene Ontology (GO) enrichment analysis indicated that the cytokines affected by ZWT treatment were primarily involved in biological processes such as cell apoptosis and inflammatory response (Fig. 3D). Furthermore, GO molecular function enrichment analysis revealed that the differentially expressed proteins were predominantly associated with the chemokine signaling pathway (Fig. 3E). Tissue injury triggers the release of chemokines, which recruit CCR2 + inflammatory macrophages and subsequently elevate circulating inflammatory cytokine levels [20]. As evidenced by ELISA assays, ZWT treatment significantly suppressed the 5/6 nephrectomy-induced elevation of CCL2 levels (Fig. 3F), and concomitantly reduced the concentrations of pro-inflammatory cytokines including IL-1β and IL-6 (Fig. 3G, H), supporting the notion that ZWT mitigates inflammatory cytokine dysregulation and modulates chemokine activity in the context of cardiorenal syndrome.

### Qualitative analysis of major components in ZWT

**Fig. 4:**
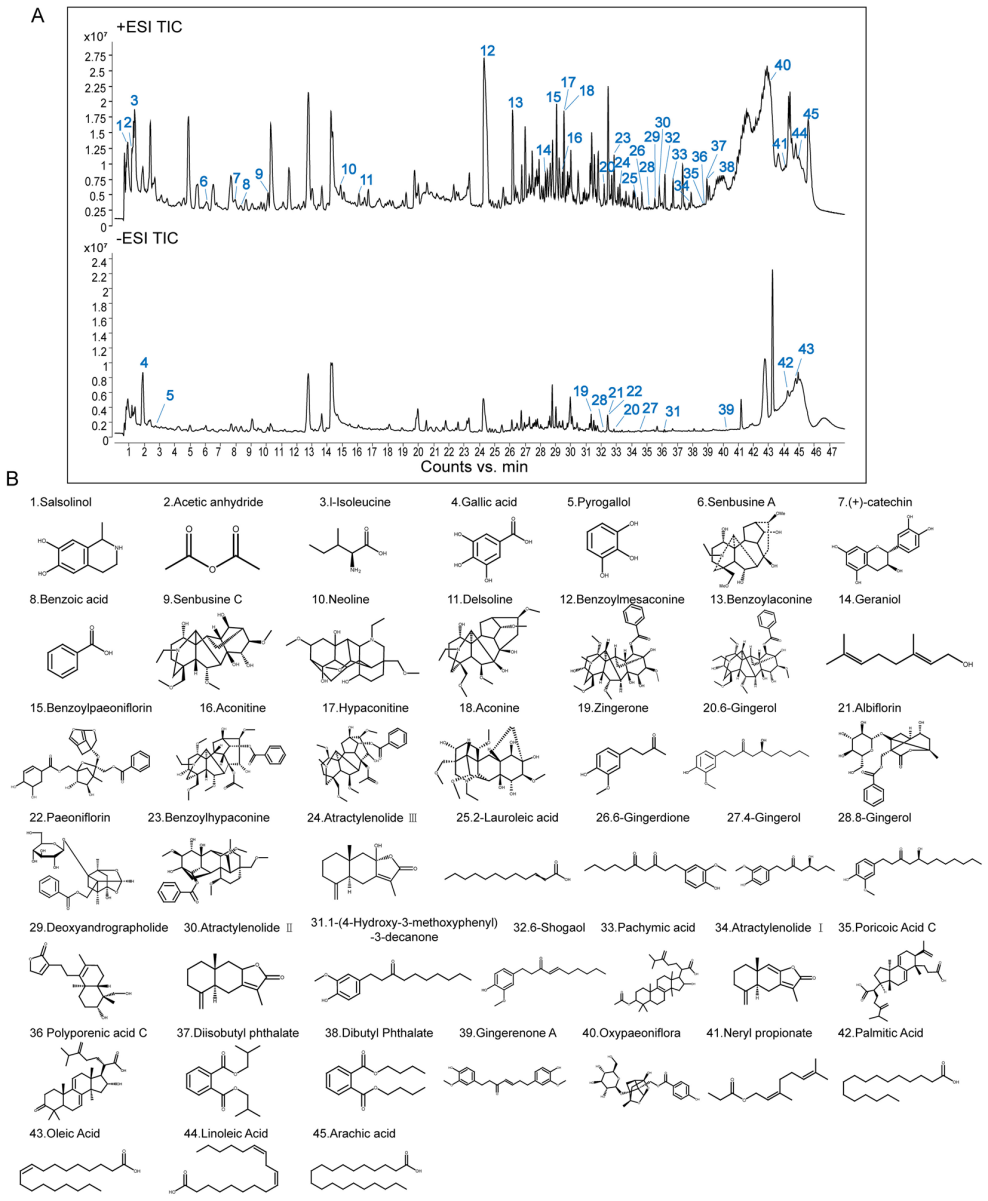
Qualitative Analysis of Major Components in ZWT. Qualitative Analysis of Major Components in ZWT. **A** Comprehensive qualitative analysis of ZWT components by UHPLC-Q/TOF–MS. Total ion chromatogram display separated constituents in positive (upper panel) and negative (lower panel) ionization modes. **B** Chemical structures of the 45 major compounds identified in ZWT

HPLC fingerprint analysis of the ZWT extract demonstrated method repeatability and manufacturing consistency. The relative standard deviations (RSDs) of both retention times (≤ 0.18%) and peak areas (≤ 1.90%) for all target peaks were well below the 2% acceptability threshold. These results confirm the robustness of the analytical method, the stability of the preparation process, and the batch-to-batch uniformity of compositional content in ZWT (Fig. S4 and Supplementary Table 1). To identify the main active constituents of ZWT, we employed a qualitative analysis using UHPLC-Q/TOF–MS. The total ion chromatogram (Fig. 4A) revealed the primary chemical constituents in the extract. Based on retention time, accurate molecular weight, fragmentation patterns, and relevant literature, a total of 45 major compounds were identified (Supplementary Table 2). These include paeoniflorin, atractylenolide III, 6-shogaol, 6-gingerol, benzoylhypaconitine, benzoylmesaconine, and hypaconitine, and others. The chemical structures of these identified compounds are shown in Fig. 4B.

### Profiling of absorbed constituents of ZWT in blood

**Fig. 5:**
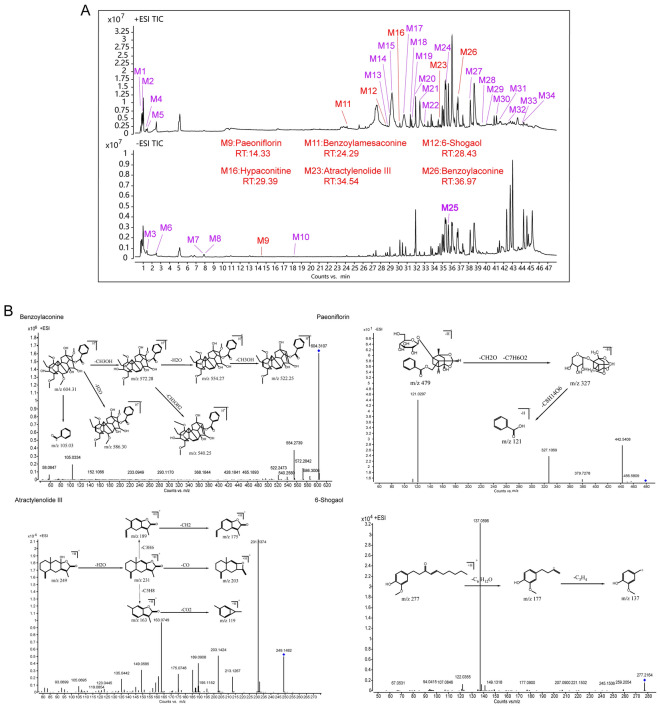
Identification of Blood-Absorbed Active Components of ZWT. Identification of Blood-Absorbed Active Components of ZWT. **A** Baseline blood (50 μl) was collected via orbital sinus puncture in rats as blank control plasma. Rats were orally administered ZWT (7 g/kg, crude drug equivalent) and plasma samples were collected at 0, 5, 15, 30 min and 1, 2, 4, 8, 12, 24 h post-ZWT administration. Post-dose plasma samples were pooled and analyzed by UHPLC-Q/TOF–MS. Total ion chromatograms are shown in positive (upper panel) and negative (lower panel) ionization modes; red labels highlight the 6 major components with their names and retention times (RT). **B** Critical cleavage pathways of four systemically absorbed components (Benzoylaconitine, paeoniflorin, atractylenolide III, and 6-Shogaol), comparing prototype compounds with their corresponding metabolites identified through pharmacokinetic profiling

Following the qualitative analysis of ZWT extract, we further investigated its bioavailable components in circulation using UHPLC-Q/TOF–MS. Under optimized chromatographic and mass spectrometric conditions, the total ion chromatogram (Fig. 5A) clearly displayed the primary ZWT-derived compounds present in the bloodstream. By determining their accurate mass and corresponding molecular formula, we identified a total of 34 components in the blood (Supplementary Table 3). Additionally, several representative compounds were selected through MS/MS fragmentation analysis to further elucidate their structural features and critical cleavage pathways (Fig. 5B).

### Bioactive components of ZWT confered protection against cardiorenal dysfunction

**Fig. 6:**
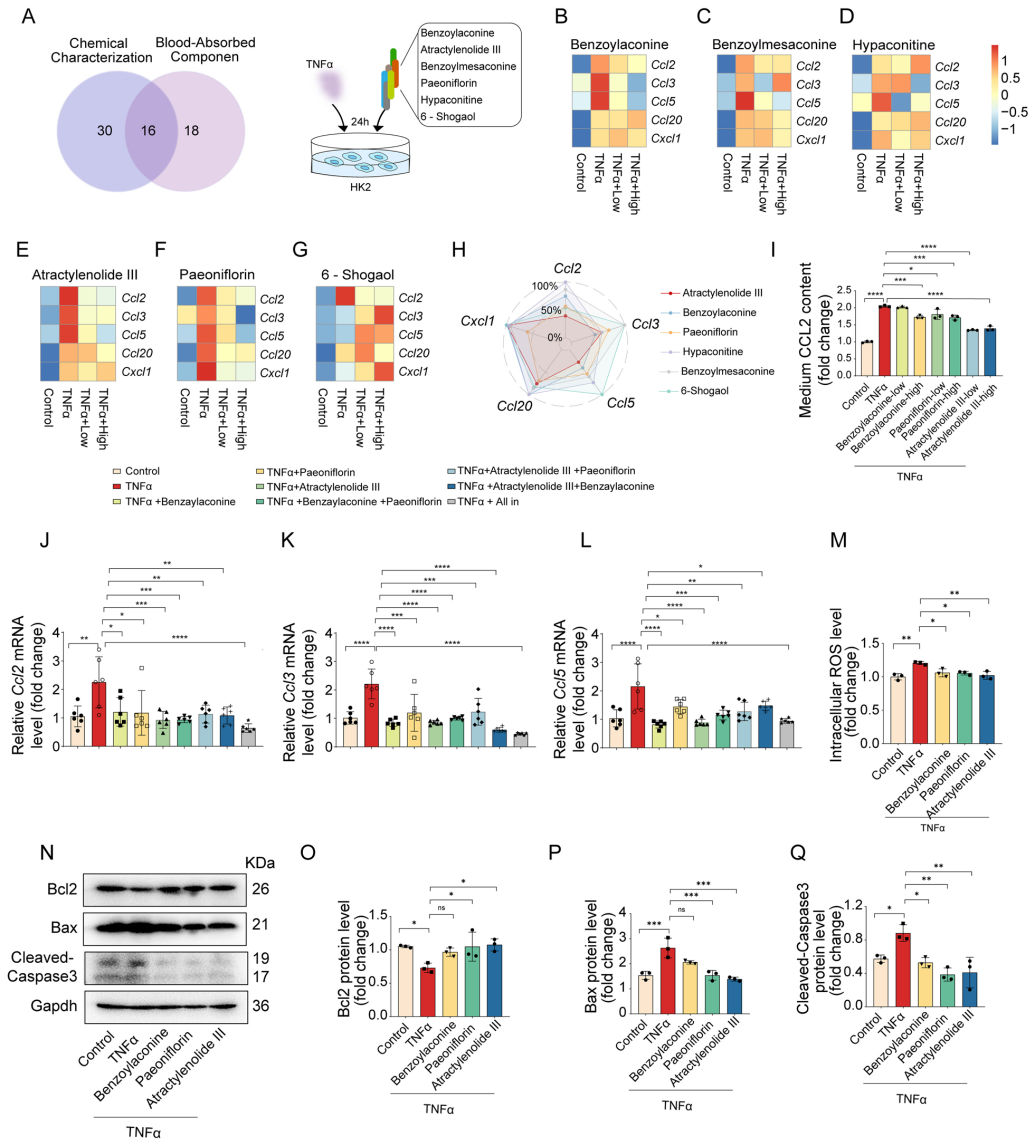
ZWT active components suppressed TNFα-induced chemokine release and cellular injury in HK-2 cells. **A** Left: Venn diagram illustrating the overlap between the chemically characterized and the blood-absorbed components of ZWT. Right: Experimental scheme for the co-treatment of HK-2 cells with TNFα 10 ng/mL and ZWT active constituents for 24 h. **B–G** RT-qPCR analysis of chemokine-related gene expression including benzoylaconine, benzoylmesaconine, and hypaconitine at (10 and 20 μM), atractylenolide III and paeoniflorin at (30 and 60 μM), and 6-shogaol at (10 and 20 μg/mL) (n = 5–6). Heatmaps represent the average expression levels. **H** Radar plot illustrating the modulation patterns of chemokines based on data from **B**–**G**. Efficacy was calculated as the ratio of TNFα + ZWT high-dose / TNFα, with proximity to the center indicating stronger normalization. **I** ELISA quantification of CCL2 secretion in HK-2 cell supernatants (n = 3). **J–L** Relative mRNA expression of CCL5, CCL3, and CCL2 in HK-2 cells treated with individual or combined monomers including benzoylaconine (10 μM), atractylenolide III (30 μM), and paeoniflorin (30 μM). **M** Representative images and quantification of intracellular ROS levels in HK-2 cells (n = 3). **N** Representative western blot images of Bcl-2, Bax, and Cleaved-Caspase3 (n = 3). **O–Q** Densitometric quantification of Bcl-2, Bax, and Cleaved-Caspase3 levels normalized to Gapdh. Data in **I–M** and **O–Q** were analyzed using one-way ANOVA with Tukey's test, with *p* ≤ 0.05 considered statistically significant. * *p* ≤ 0.05, ** *p* < 0.01, *** *p* < 0.001, **** *p* < 0.0001

Chemokines play a critical role in recruiting CCR2 + inflammatory macrophages and elevating circulating cytokine levels during tissue injury [21]. Building on our observation of significant TNFα upregulation in uremic kidneys (Fig. S1F), we established a TNFα-induced injury model in HK-2 renal tubular epithelial cells to explore the regulatory effects of ZWT. Six compounds were prioritized from the 16 overlapping prototypes for validation (Fig. 6A): paeoniflorin, benzoylmesaconine, 6-shogaol, hypaconitine, atractylenolide III, and benzoylaconine. They were selected based on their relevance to the inflammatory/chemokine pathways altered by ZWT in our proteomics study (Fig. 3) and their established bioactivity [22–27]. The compound concentrations used were based on literature-established safe and bioactive ranges [22, 28, 29]. Transcriptional analysis revealed that benzoylaconine, atractylenolide III, and paeoniflorin significantly inhibited the TNFα-induced expression of multiple chemokines including CCL2, CCL3, CCL5, CCL20, and CXCL1 (Fig. 6B–G). Radar chart analysis further demonstrated that these compounds exhibited varying efficacy across different chemokines, with the most pronounced effects observed on CCL2 and CCL5 (Fig. 6H). ELISA quantification confirmed that the three most potent inhibitors—benzoylaconine, paeoniflorin, and atractylenolide III—significantly suppressed TNFα-induced CCL2 secretion (Fig. 6I). Furthermore, the triple combination of these compounds produced a significantly stronger inhibition of CCL2, CCL3, and CCL5 than any individual or pairwise treatment, demonstrating a clear synergistic effect at the transcriptional level (Fig. 6J–L).

We subsequently investigated whether ZWT’s active components counter oxidative stress, another key mediator of renal damage. The results showed that the active ingredients of ZWT effectively mitigated the TNFα-induced oxidative burst in HK-2 cells (Fig. 6M). This antioxidant activity was coupled with a potent inhibition of apoptosis, as evidenced by the downregulation of Bcl-2 and the concomitant upregulation of Bax and Cleaved-Caspase 3 (Fig. 6N–Q). Collectively, these findings indicated that the core components of ZWT exerted protective roles by simultaneously suppressing chemokine-mediated inflammation and bolstering cellular resilience against oxidative and apoptotic stress.

To dissect the specific roles of the key ZWT bioactives, we evaluated them individually in the UC mouse model. At pharmacologically validated doses, benzoylaconine, paeoniflorin, and atractylenolide III all attenuated renal pathology, as seen in reduced tubular injury, fibrosis (Fig. S5A), and *Kim1* expression (Fig. S5B). Paeoniflorin and atractylenolide III further suppressed renal *Ccl2* (Fig. S5C). Assessment of cardiac endpoints revealed a spectrum of activity. Benzoylaconine primarily enhanced systolic function and ventricular remodeling, with a moderate impact on hypertrophy (Fig. S5D–I). In contrast, paeoniflorin and atractylenolide III exhibited stronger anti-hypertrophic effects, the latter also improving systolic function and reducing ventricular mass (Fig. S5D-K). These findings suggest complementary mechanisms underlie the integrated cardiorenal protection of the ZWT formulation.

### ZWT attenuates chemokine production by inhibiting NF-κB signaling activation

To systematically investigate the mechanisms by which ZWT modulated chemokine signaling in UC, we conducted an integrated network pharmacology approach. Potential therapeutic targets of ZWT were cross-referenced with UC-related genes from public databases, yielding 74 overlapping targets identified via Venn analysis (Fig. S6A). GO and KEGG enrichment analyses indicated that ZWT might influence chronic inflammation and ROS metabolic disorders, with the NF-κB signaling pathway identified as a prominent candidate (Fig. S6B, C). We first validated these predictions in vitro using HK-2 cells. Western blot analysis showed that the three bioactive compounds significantly inhibited TNFα-induced phosphorylation of NF-κB p65, while total p65 levels remained unchanged (Fig. S6D-G). Consistent with these in vitro findings, the inhibitory effect of ZWT on the NF-κB pathway was further confirmed in the 5/6 nephrectomy-induced UC model. ZWT treatment significantly suppressed p65 phosphorylation and attenuated the degradation of IκBα and IKKβ in renal tissues (Fig. S6H-L). To elucidate the structural basis for this inhibition, we performed molecular docking of the core ZWT components against key upstream kinases of the NF-κB pathway using AutoDock Vina. A binding affinity heatmap revealed strong interactions for multiple components, with more negative scores indicating higher affinity (Fig. S6M). Notably, paeoniflorin and atractylenolide III exhibited robust binding within the catalytic pockets of IKKβ and GSK3β, respectively (Fig. S6N, O). Stable hydrogen bonds with key amino acid residues at these sites provide a plausible structural mechanism for the observed suppression of NF-κB cascade phosphorylation. These computational modeling data suggest that the suppression of NF-κB pathway activation and downstream CCL2 production by ZWT may be mediated, at least in part, through direct interaction with these kinase targets.

### ZWT mitigated macrophage-mediated inflammatory responses and cardiomyocyte hypertrophy

**Fig. 7:**
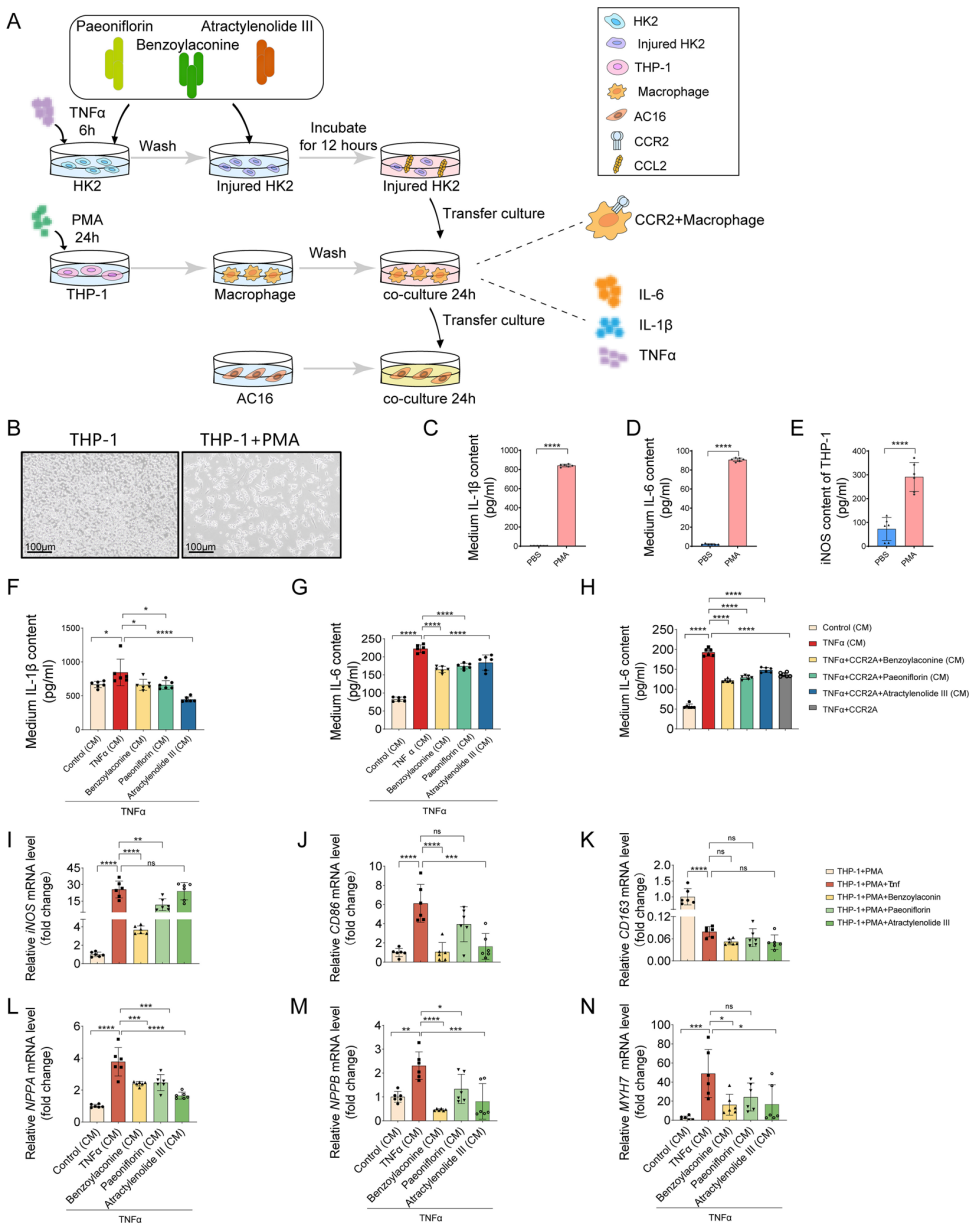
ZWT Mitigated Macrophage-Mediated Inflammatory Responses and Cardiomyocyte Hypertrophy. ZWT Mitigated Macrophage-Mediated Inflammatory Responses and Cardiomyocyte Hypertrophy. **A** Schematic of a three-stage cell culture model. HK-2 cells were pretreated with TNFα in the presence or absence of benzoylaconine (20 μM), paeoniflorin (60 μM) and atractylenolide III (60 μM) for 6 h. After TNFα washout, these ingredients were reapplied, and HK-2 equilibrated for 12 h before supernatant collected. Macrophages pre-stimulated with PMA (10 ng/mL, 24 h) were cultured with HK-2-conditioned supernatants for 24 h. Resultant macrophage supernatants were then used to treat AC16 cardiomyocytes for 24 h prior to functional assays. **B** Phase-contrast micrographs of THP-1 cells: (Left) untreated cells exhibiting a round, suspension morphology; (Right) cells treated with PMA, showing adherent growth and an irregular morphology. Scale bar = 100 μm. **C–E** ELISA quantified THP-1 macrophage differentiation markers, n = 6. Data in **C–E** were analyzed by unpaired t-test. **F–G** IL-1β and IL-6 levels in macrophage media after exposure to monomer-treated HK-2 supernatants, measured by ELISA, n = 6. **H** IL-6 secretion levels in macrophage culture media were measured after treatment with 10 μM CCR2 antagonist 4 (CCR2A), followed by administration of three monomeric compounds. n = 6 samples per group. **I–K** Relative mRNA levels of M1 markers (*iNOS*, *CD86*) and M2 marker (*CD163*) in THP1 cells, normalized to 18S rRNA, n = 3 samples per group. **L–N** Relative mRNA levels of *NPPA*, *NPPB*, and *MYH7* in AC16 cells, normalized to *GAPDH*, n = 3 samples per group, Data in **F–N** were analyzed by one-way ANOVA with Tukey's test. Data in **C–N** were presented as mean ± SD, with* p* ≤ 0.05 considered statistically significant. *p ≤ 0.05, **p < 0.01, ***p < 0.001, ****p < 0.0001

To confirm that ZWT alleviates cardiac injury by suppressing chemokine release from renal tubular epithelial cells and reducing macrophage-mediated inflammatory cytokine secretion, we developed a three-stage cell culture model. First, HK-2 cells were exposed to TNFα in the presence or absence of benzoylaconine, paeoniflorin, or atractylenolide III for 6 h, after which the TNFα was removed. The cells were incubated with or without these compounds for 12 h before supernatant collection (Fig. 7A). Second, to induce the polarization of THP-1 into CCR2 + proinflammatory macrophages [30], THP-1 cells were stimulated with PMA for 24 h [31] (Fig. 7B–E). These macrophages were then incubated with the conditional medium from TNFα injured HK-2 cells for an additional 24 h (Fig. 7A). Finally, the supernatant from these macrophages was collected and used to culture AC16 cardiomyocytes (Fig. 7A). ELISA analysis revealed that macrophages exposed to conditioned medium from compound-treated HK-2 cells secreted significantly lower levels of inflammatory cytokines (Fig. 7F, G). Notably, this anti-inflammatory effect was abolished when macrophages were pre-treated with the CCR2 antagonist for 24 h, demonstrating that the benefits of these compounds are indeed dependent on CCR2 signaling (Fig. 7H). To further characterize macrophage polarization patterns, we performed mRNA expression analysis in macrophages exposed to conditioned medium from compound-treated HK-2 cells. TNFα stimulation significantly increased the expression of the M1 markers *iNOS* and *CD86*. Treatment with benzoylaconine notably reduced both *iNOS* and *CD86* levels, while paeoniflorin and atractylenolide III each selectively decreased *iNOS* or *CD86*, respectively. None of the three compounds significantly increased the expression of the M2 marker *CD163* (Fig. 7I–K). Additionally, AC16 cardiomyocytes cultured in conditioned medium from these macrophages exhibited reduced expression of hypertrophy-related markers, including *NPPA*, *NPPB*, and *MYH7*, indicating a protective effect against cardiomyocyte hypertrophy (Fig. 7L–N). In summary, these findings suggest that ZWT attenuates kidney-derived chemokine-driven inflammation by modulating the macrophage inflammatory response, ultimately leading to improved cardiomyocyte function.

### Neutralization of systemic CCL2 recapitulated the cardiorenal protection of ZWT

**Fig. 8:**
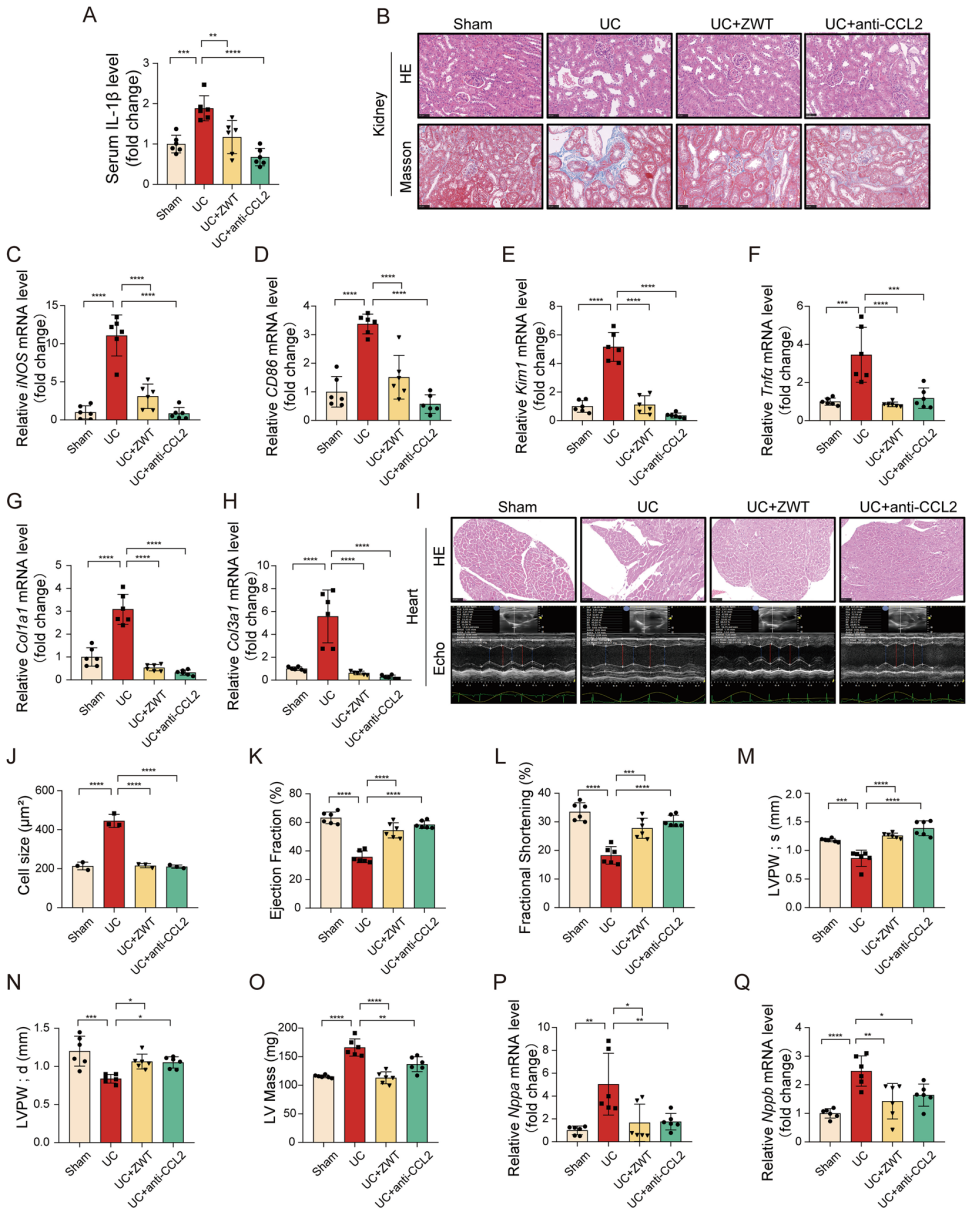
CCL2 Blockade Attenuates Cardiorenal Injury in Uremic Cardiomyopathy. Mice were subjected to 5/6 nephrectomy and treated with either ZWT (10 g/kg/day by intragastric administration) or an anti-CCL2 antibody (10 mg/kg every 10 days by intraperitoneal injection) for 8 weeks. **A** Circulating level of IL-1β was measured by ELISA; n = 6 per group. **B** Representative images of kidney sections stained with hematoxylin–eosin (HE) and Masson (scale bar = 50 μm; n = 3 mice per group). **C, D** Relative mRNA expression of M1 macrophage polarization markers in kidney tissue was determined by qPCR; n = 6 per group. **E–H** Relative mRNA levels of kidney injury, inflammation, and fibrosis markers were assessed by qPCR. **I** Representative images of HE-stained heart sections (n = 3 mice per group) and M-mode echocardiography (n = 6 mice per group). **J** Quantification of cardiomyocyte cross-sectional area from HE-stained sections (n = 3 mice per group). **K–O** Echocardiographic analysis included ejection fraction (EF), fractional shortening (FS), left ventricular posterior wall thickness at end‑systole (LVPW; s), left ventricular posterior wall thickness at end‑diastole (LVPW;d), and left ventricular mass (LV Mass); n = 6 mice per group. **P**, **Q** Relative mRNA expression of *Nppa* and *Nppb* in heart tissue normalized to 18S rRNA; n = 6 per group. Data were presented as mean ± SD and were analyzed by one‑way ANOVA with Tukey’s test. *p* ≤ 0.05 considered statistically significant. *p ≤ 0.05, **p < 0.01, ***p < 0.001, ****p < 0.0001

To verify whether the suppression of CCL2 was a primary driver of the observed therapeutic benefits, a CCL2 neutralizing antibody (anti-CCL2) was administered to UC mice, with the ZWT serving as a parallel positive control. Neutralization of CCL2 significantly reduced systemic inflammatory markers; serum IL-1β level decreased similarly in the anti-CCL2 and ZWT-treated groups (Fig. 8A). Histological analysis via HE and Masson staining revealed that CCL2 neutralization significantly attenuated renal structural damage and interstitial fibrosis (Fig. 8B). This was accompanied by a marked reduction in the infiltration of renal M1 macrophages, as evidenced by the downregulated expression of *iNOS* and *CD86* (Fig. 8C, D). Furthermore, anti-CCL2 treatment effectively lowered the mRNA levels of renal injury markers, including *Kim1* and *Tnfα* (Fig. 8E, F), and suppressed the expression of pro-fibrotic genes *Col1a1* and *Col3a1* (Fig. 8G, H). These results demonstrated that blocking the CCL2 axis achieved a renoprotective profile similar to that of ZWT. Histological and echocardiographic analyses showed that anti-CCL2 treatment alleviated structural injury (Fig. 8I) and significantly reduced cardiomyocyte cross-sectional area (Fig. 8J). Echocardiography confirmed the restoration of systolic function, as indicated by increased ejection fraction and fractional shortening (Fig. 8K, L). The treatment also prevented adverse ventricular remodeling, stabilizing left posterior wall thickness (Fig. 8M–O). These structural and functional improvements were further supported by significant downregulation of the cardiac stress markers *Nppa* and *Nppb* (Fig. 8P, Q). CCL2 neutralizing antibody attenuated cardiorenal injury in UC mice, phenocopying the effects of ZWT, suggesting that CCL2 is a critical mediator in this pathological process.

## Discusion

**Fig. 9:**
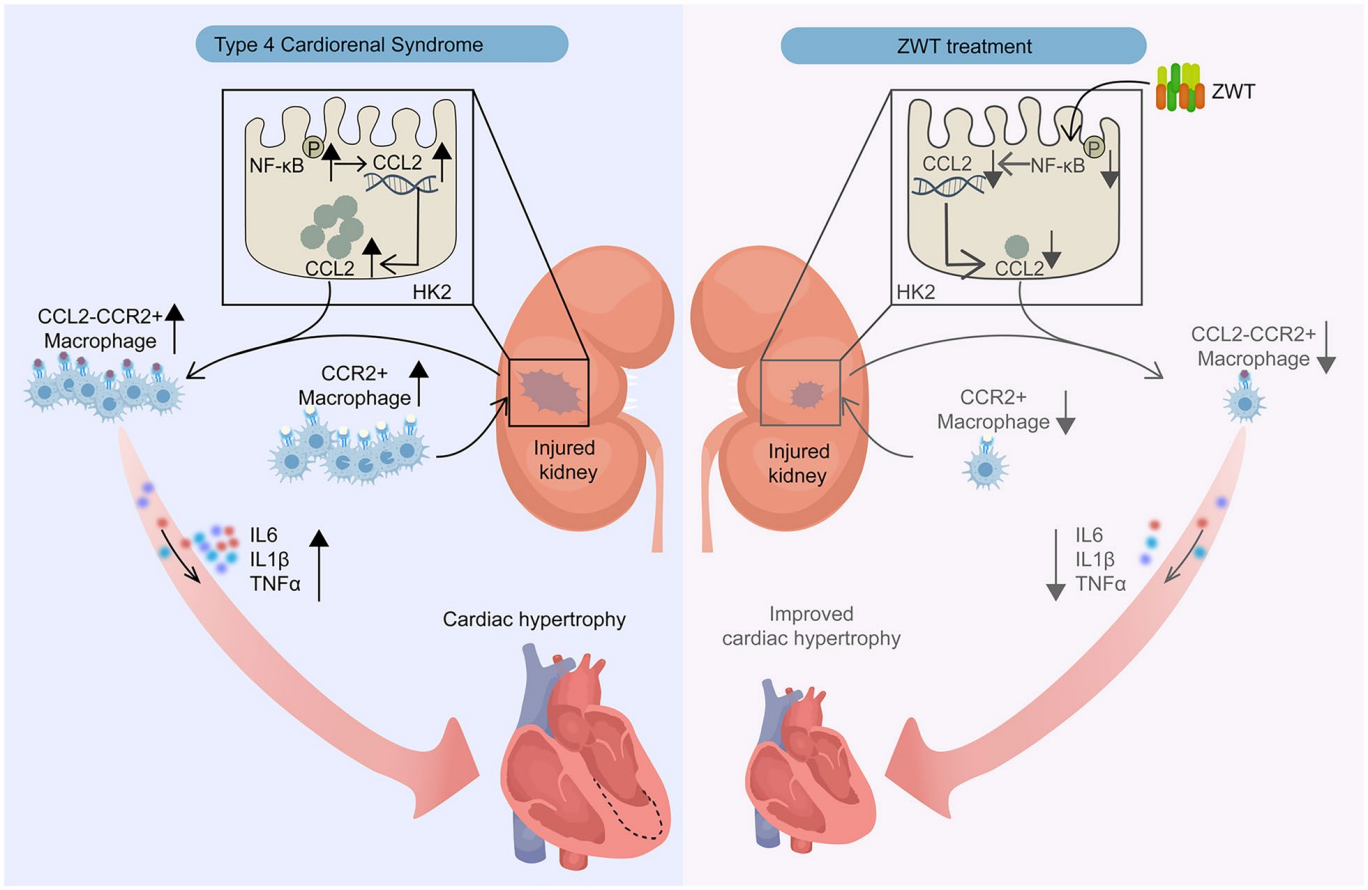
ZWT Blocked Pathological Cross-Talk in UC. Proposed mechanisms of ZWT in mitigating UC and renal dysfunction. ZWT suppressed TNFα-induced chemokine release in renal tubular epithelial cells and mitigated CCR2 + macrophage recruitment. These effects led to reduced levels of inflammatory cytokines in the blood and ultimately alleviated cardiomyocyte hypertrophy

This study demonstrated that ZWT exerts dual cardiorenal protective effects in a UC mouse model. ZWT improved cardiac and renal function, and reduced circulating inflammatory cytokines and chemokines. Mechanistically, ZWT suppressed TNFα-induced chemokine release in renal tubular epithelial cells, decreased CCR2 + macrophage-mediated inflammation and alleviated cardiomyocyte hypertrophy (Fig. 9). Our findings uncover a previously unrecognized mechanism by which ZWT targets the kidney-heart inflammatory axis, offering a comprehensive therapeutic strategy of "cardio-renal simultaneous intervention” through coordinated regulation of interorgan communication.

Cross-organ communication is primarily mediated by secreted proteins, metabolites, and other signaling molecules, which coordinate physiological and pathological processes between distant organs. While previous studies reported ZWT's protective effects on single organs [32], our study uniquely explores its role in modulating the systemic kidney-heart interplay. In this study, proteomic profiling revealed that ZWT administration significantly reduces circulating IL-6 and IL-1β levels. These inflammatory mediators are known to modulate immune landscapes across distant organ systems [33]. Our findings thus outline the molecular basis underlying the synergistic cardiorenal protection and multi-organ efficacy of ZWT.

The recruitment and activation of macrophages at the injury site represent a pivotal link in the inflammatory crosstalk between the kidney and heart. In this study, we found that ZWT exerts its renoprotective effects by transcriptionally regulating CCL2 expression, thereby reducing macrophage chemotaxis and M1 polarization. Crucially, direct in vivo neutralization of CCL2 significantly suppressed renal M1 macrophage polarization and ameliorated both renal injury and cardiac hypertrophy. This neutralization effectively phenocopies the therapeutic effects of ZWT, thereby supporting that the CCL2/CCR2-mediated kidney-heart inflammatory axis serves as a predominant mechanism in UC.

Previous studies reported that benzoylaconine, paeoniflorin, and atractylenolide III possess individual cardioprotective or renoprotective properties [29, 34, 35]. Consistent with these properties, we observed that these compounds directly attenuated hypertrophy in AC16 cardiomyocytes (Fig. S7A, B). Furthermore, we extended these findings by demonstrating that their co-administration synergistically inhibits pro-inflammatory chemokines in HK-2 cells (Fig. 6J–L). In vivo, significant suppression of renal CCL2 was specific to paeoniflorin and atractylenolide III. Benzoylaconine primarily enhanced systolic function, whereas the other two compounds showed stronger anti-hypertrophic efficacy. This functional divergence may stem from their varying affinities for upstream signaling targets, as suggested by our docking analysis. Such specialization likely underlies the synergistic effect observed with their combination, illustrating how ZWT achieves integrated cardiorenal protection through multi-target coordination.

The promising therapeutic potential of ZWT is further supported by the established pharmacokinetic profiles of its key constituents and its long-term safety. In this study, 8 weeks of ZWT treatment showed no hepatotoxicity or overt systemic side effects (Fig. S3A), consistent with clinical meta-analyses reporting favorable tolerability in patients [36, 37]. However, detailed systemic pharmacokinetics and tissue-specific pharmacodynamics remain to be fully elucidated to further validate its clinical applicability in chronic uremic cardiomyopathy.

Despite these insights, several limitations remain. Although our in vitro models offer mechanistic clarity, they cannot fully recapitulate the systemic complexity of the cardio-renal axis in vivo. Therefore, large-scale randomized controlled trials are needed to validate the long-term clinical efficacy of ZWT. In addition, CCL2 overexpression and targeted NF-κB inhibition strategies are warranted to further substantiate the necessity of the NF-κB–CCL2 axis in mediating the therapeutic effects of ZWT.

Collectively, these findings reveal that ZWT protects against uremic cardiomyopathy by targeting the kidney-heart inflammatory axis and suppressing CCL2/CCR2-mediated macrophage activation. This study offers a robust mechanistic foundation for its therapeutic application in cardiorenal syndrome.

## Methods

### Preparation of ZWT

ZWT comprises five herbs at a ratio of 3:2:3:3:3: Aconiti Lateralis Radix Praeparata (processed lateral root; batch No. 200201), Atractylodes macrocephala Koidz. (raw rhizome; batch No. 210125), Poria cocos (raw sclerotium; batch No. 210424), Paeoniae Radix Alba (raw root; batch No. 201001), and Zingiberis Rhizoma (raw rhizome; batch No. 200601). Among them, only Aconiti Lateralis Radix Praeparata was used as a processed material, while the other four were used in raw form. All crude herbs were obtained from Wansheng Traditional Chinese Medicine Decoction Pieces Co., Ltd. (Anhui, China), and all materials were authenticated in accordance with the Chinese Pharmacopoeia (2025 edition). For decoction preparation, the mixed herbs were soaked in a tenfold weight of water for 12 h and then boiled for 1 h. The decoction was then filtered. Subsequently, 8 times the weight of water was added and decocted for 30 min. The two batches of decoction were combined and concentrated to a final concentration of 1 g/mL using a rotary evaporator, then stored at 4 °C.

### Qualitative identification of the constituents of ZWT

The extracts were dried in an oven at 65 °C. A 20 mg sample of ZWT was dissolved in 50% methanol and sonicated for 30 min. The filtrate was passed through a 0.22 μm microporous membrane to obtain the test solution. Chromatographic separation of ZWT was performed using an Agilent 1290 Infinity II ultra-performance liquid chromatography (UPLC) system, with a ZORBAX Eclipse Plus C18 column (1.8 μm, 3.0 × 150 mm, Agilent, USA). The mobile phases were designated as phase A and phase B. Phase A was an ultrapure aqueous solution containing 0.1% formic acid and phase B was an acetonitrile solution with 0.1% formic acid. The mobile phase composition was consistent in both positive and negative ion modes. The elution gradient was as follows: 0–8 min, 5%-9% B-phase; 8–16 min, 9%-15% B-phase; 16–24 min, 15%-22% B-phase; 24–44 min, 22%-95% B-phase; 44–48 min, 95–5% B-phase. The column temperature was maintained at 30 °C with a flow rate of 0.3 mL/min and an injection volume of 5 μL. The post-run time was set to 2 min. Mass spectrometric analysis of ZWT was performed using an Agilent 6545A quadrupole time-of-flight mass spectrometer. The electrospray ionization (ESI) source was operated at 320 °C, with a drying gas flow rate of 5 L/min (nitrogen), a spray nozzle pressure of 20 psig, and a sheath gas (nitrogen) at 350 °C with a flow rate of 11 L/min. The capillary voltage was set to 3500 V, the skimmer voltage to 65 V, and the fragmentor voltage to 120 V. Both positive and negative ion modes were used for ion source operation. Mass spectra were collected within the range of 60–1700 daltons, with a sweep rate of 1.5 maps/s. For secondary mass spectrometry acquisition, the injection volume was 10 μL, and the collision voltage was applied in three gradients: 10 V, 20 V, and 40 V.

### HPLC fingerprint analysis of ZWT

ZWT was prepared in accordance with the procedure described in Sect. "Preparation of ZWT". Chromatographic analysis was performed under the conditions outlined in Sect. "Qualitative identification of the constituents of ZWT". The HPLC fingerprint was recorded at a detection wavelength of 280 nm. Characteristic peak retention times and relative peak areas were used as key parameters for quality assessment.

### Animals

Male C57BL/6J mice and male SD rats were purchased from GemPharmatech Co., Ltd. The mice were aged 8–10 weeks and weighed 25–28 g, while the rats were aged 8–10 weeks and weighed 200–220 g. Animals were housed in the Center for Experimental Animals at China Pharmaceutical University, Nanjing, China. The feeding condition is constant temperature (25 ± 1 °C) and constant humidity (70%). Animals were maintained on a 12-h light–dark cycle and had access to water and food ad libitum, with a standard chow diet provided. All procedures involving experimental animals were conducted in accordance with protocols approved by the Committee for Animal Research of China Pharmaceutical University (Ethics No. 2025-01-062) and adhered to the Guidelines for the Care and Use of Laboratory Animals.

### Preparation of drug-containing plasma in rats

The rats were fasted without food for 12 h. Blood samples were collected as baseline controls before administration. Mice were then orally gavaged with ZWT at a dose equivalent to 7 g/kg crude herbs. The prepared decoction was concentrated to 1 g/mL (crude-herb equivalent), and the administration volume was therefore 7 mL/kg. Blood samples were subsequently collected from the orbital sinus at designated time points: 0 min, 5 min, 15 min, 30 min, 1 h, 2 h, 4 h, 8 h, 12 h, and 24 h post-administration. The collected blood samples were centrifuged at 3000 rpm for 10 min to separate plasma, and equal volumes of plasma from each time point were pooled before storage at -80 °C for further analysis.

### Qualitative identification of the plasma components of ZWT

A non-target sample was prepared using rat serum, with an initial injection volume of 2 μL and a secondary injection volume of 8 μL. The mobile phases were designated as phase A and phase B, with phase A being an ultrapure aqueous solution containing 0.1% formic acid and phase B being pure acetonitrile. The composition of the mobile phases was the same for both positive and negative ion modes. The elution gradient, flow rate, and mass spectrometry conditions were identical to those used for the separation of the herbal components.

### Animal models

Mice were anesthetized with tribromoethanol (T161626, Aladdin, China), prepared as a 1.25% (w/v) working solution and administered via intraperitoneal injection at a dose of 250 mg/kg, with the injection volume adjusted according to individual body weight. Uremia was induced via a two-step 5/6 nephrectomy: approximately two-thirds of the left kidney was resected after renal pedicle ligation, followed by complete right nephrectomy 7 days later. Sham-operated mice underwent laparotomy and suturing without kidney removal. Cardiac function was evaluated by echocardiography at the study endpoint (8 weeks post-surgery). Successful induction of myocardial injury was defined as a reduction in left ventricular ejection fraction (EF) below 55%.

Efficacy Assessment of ZWT: Mice were randomized into six groups: sham, model, ZWT low-dose (5 g/kg/d), ZWT high-dose (10 g/kg/d), Captopril (9.75 mg/kg/d; CAS: 62571‑86‑2, MCE, USA), and Finerenone (10 mg/kg/d; Kerendia, BAYER, BXK01J1). Treatments were administered intragastrically once daily for 8 weeks.

Efficacy Assessment of Bioactive Components of ZWT: Mice were randomized into six groups: sham, model, ZWT (10 g/kg/d), Benzoylaconine (10 mg/kg/d; A0631, Chengdu Must Biotechnology), Atractylenolide III (20 mg/kg/d; A0374, Chengdu Must Biotechnology), Paeoniflorin (50 mg/kg/d; A0133, Chengdu Must Biotechnology). All compounds were given intragastrically once daily for 8 weeks.

CCL2 Blockade Experiment: Following 5/6 nephrectomy, mice were treated after 8 weeks with either ZWT intragastrically (10 g/kg/day) or anti‑CCL2 neutralizing monoclonal antibody (Selleck, clone 2H5, A2132) at a dose of 10 mg/kg every 10 days, for a duration of 8 weeks. Sham and model mice received vehicle treatments matched to the corresponding intervention arms in volume, frequency, and administration route: daily oral gavage with double-distilled water (matching the ZWT group) and intraperitoneal vehicle injection every 10 days (matching the anti-CCL2 group).

### Echocardiography

Cardiac function was assessed using echocardiography (Vevo 3100LT, China Pharmaceutical University). After aligning the transverse B-mode with the papillary muscles, systolic function was measured using M-mode images. Briefly, the mice were anesthetized with isoflurane (3% for induction and 1–1.5% for maintenance to maintain a heart rate between 400 and 500 beats per min) mixed with 1 L/min oxygen delivered via a facemask. Modified parasternal long-axis and short-axis views were used to acquire both two-dimensional and M-mode images. Left ventricular chamber size and wall thickness were measured from at least three beats in each projection and averaged. The following parameters were measured: left ventricular posterior wall diameter during diastole and systole (LVPW; d and LVPW; s), cardiac output as well as fractional shortening (FS%) and ejection fraction (EF%). All echocardiographic data were analyzed using Vevo Lab software.

### Histology

Heart, kidney, and liver tissue samples were fixed in 4% paraformaldehyde (P6148, Sigma-Aldrich, USA) for 24 h, followed by dehydrated and embedded in paraffin. The paraffin-embedded tissues were sectioned into 5–8 μm thick slices. These sections were then deparaffinised and stained with hematoxylin–eosin (HE) and Masson's trichrome stains respectively. Following staining, the sections were dehydrated, cleared, and mounted with neutral gum to complete the process. In immunohistochemistry experiments, sections were incubated with 3% H₂O₂ solution (diluted with distilled water) to inhibit endogenous peroxidase activity, and then washed and separated with PBS. Subsequently, the sections were incubated overnight with F4/80 polyclonal antibody (28463-1-AP, Proteintech, USA) and subsequently washed with PBS and incubated with peroxidase coupled goat anti-rabbit IgG (SA00001-2, Proteintech, USA) as secondary antibody for 60 min at room temperature and counterstained with hematoxylin. Secondary antibody incubation was then performed using a labelled antibody matched to the primary antibody, incubated for 60 min at room temperature and washed with PBS to enhance the signal and remove unbound antibody. HE staining was performed on the heart, kidney, and liver to assess the morphology. Masson’s trichrome staining was used on the heart and kidneys to evaluate fibrosis. F4/80 staining was conducted on the kidneys to assess the number of macrophages using antibody.

### RNA extraction and quantitative real-time PCR

Total RNA was extracted from tissues and cells using TRIzol reagent (R701-02, Vazyme Biotech, China) according to the manufacturer’s instructions. DNA was then reverse transcribed into cDNA using the cDNA Reverse Transcription Kit (11141ES60, Yeasen biotech, China). Real-time quantitative PCR was performed using the Hieff qPCR SYBR Green Master (Low Rox) kit (11202ES08, Yeasen biotech, China). Gene quantification was based on the difference in quantification cycles (ΔΔCt), with normalization to reference genes. he sequences of the primers utilized are listed as follows:

**Table:** 

Kim1 (mouse)	Forward	GCTGCTACTGCTCCTTGTGA
	Reverse	GGAAGGCAACCACGCTTAGA
Tnfα (mouse)	Forward	AGACAGAGGCAACCTGACCAC
	Reverse	GCACCACCATCAAGGACTCAA
Il-1β (mouse)	Forward	GGTAAGTGGTTGCCCATCAGA
	Reverse	GCTGCTCAGGGTCACAAGAAA
Col1a1 (mouse)	Forward	CTGACGCATGGCCAAGAAGA
	Reverse	CGTGCCATTGTGGCAGATAC
Col3a1 (mouse)	Forward	TGACTGTCCCACGTAAGCAC
	Reverse	GGAGGGCCATAGCTGAACTG
Nppa (mouse)	Forward	TGTACAGTGCGGTGTCCAAC
	Reverse	CGAAGCAGCTGGATCTTCGT
Nppb (mouse)	Forward	TTGAAGGACCAAGGCCTCAC
	Reverse	CGTTACAGCCCAAACGACTG
18S rRNA (mouse)	Forward	CGGCTACCACATCCAAGGAA
	Reverse	GCTGGAATTACCGCGGCT
iNOS (mouse)	Forward	GGATCCAGTGGTCCAACCTG
	Reverse	GCACATCAAAGCGGCCATAG
CD86 (mouse)	Forward	GATCAAGGACATGGGCTCGT
	Reverse	TAGGTTTCGGGTGACCTTGC
CCR2 (mouse)	Forward	CCTCAGTTCATCCACGGCAT
	Reverse	AGGGAGTAGAGTGGAGGCAG
Myh7 (human)	Forward	GAAGGACGAGGAGATGGAACA
	Reverse	GCTCTGGAGGCTCTTGACTT
Nppa (human)	Forward	AACGCAGACCTGATGGATTTC
	Reverse	AGGGCACCTCCATCTCTCT
Nppb (human)	Forward	GTTCAGCCTCGGACTTGGA
	Reverse	GCAGGGTGTAGAGGACCATTT
GAPDH (human)	Forward	GTCAAGGCTGAGAACGGGAA
	Reverse	AAATGAGCCCCAGCCTTCTC
iNOS (human)	Forward	AGGTCCAAATCTTGCCTGGG
	Reverse	ATCTGGAGGGGTAGGCTTGT
CD86 (human)	Forward	CTTCCTGCTCTCTGCTAACTTC
	Reverse	CCGCGTCTTGTCAGTTTCCA
CD163 (human)	Forward	GGGAAGGTGTGTGACAGAGG
	Reverse	GGAATGTCCCCTCCAACCAG

### Western blot

Total proteins were extracted from cultured cells. Briefly, proteins were separated by SDS-PAGE and transferred to PVDF membranes (EMD Millipore), followed by overnight incubation with appropriate primary antibodies at 4 °C. The membranes were then washed and incubated with secondary antibodies for 1 h. Protein detection was performed using an automatic chemiluminescent imaging system (Tannon-5200).

The primary antibodies used for western blotting are listed as follows: GAPDH (10494-1-AP, 1:1000, Proteintech, China), NF-κB p65 (8242T, 1:1000, CST, USA), Phospho-NF-κB p65 (3033T, 1:1000, CST, USA). IκBα (A19714, 1:1000, ABclonal, China), Phospho-lκBα-S32 (AP0707, 1:1000, ABclonal, China), IKKβ (A2087, 1:1000, ABclonal, China), Phospho-lKKα-S180 (AP0506, 1:1000, ABclonal, China). The secondary antibodies used for western blotting are listed as follows: HRP-labeled Goat Anti-Rabbit IgG (A0208, 1:5000, Beyotime, China); HRP-labeled Goat Anti-Mouse IgG (A0216, 1:5000, Beyotime, China).

### Proteomics

Mouse serum samples were randomized using Excel's RAND function and the samples were distributed randomly into 96-well plates. Based on the randomization results, 10 µL of each sample was added to the plate with control samples placed in specific wells. A hybridization mix was prepared and added to the 96-well plate, with 1 µL of sample per well. The plate was incubated at 4 °C for 24 h. Set the PCR programme for extension and amplification: 50 °C for 20 min; 95 °C for 5 min; 95 °C for 30 s, 54 °C for 1 min, 60 °C for 1 min for a total of 17 cycles; and a final 10 °C hold. Press the pause button after running the PCR instrument to keep the temperature at 50 °C. After microarray pre-treatment, the detection solution and amplification products were added for onboarding. Finally, the data file was exported, analyzed and the result document was generated using Olink NPX software.

### Serum assays

Serum creatinine and urea nitrogen levels were measured using commercial kits. The creatinine assay (E-BC-K188-M, Elabscience, China) was based on the creatine oxidase method. The blood urea nitrogen test (BC1535, Solarbio, China) was based on the urease method. The absorbance of the indophenol was measured at 580 nm. ELISA was used to detect serum IL-6 (431304, Biolegend, USA), IL-1β (E-EL-M0037, Elabscicence, China) and CCL-2 (432704, Biolegend, USA) levels. The samples were added to an ELISA plate pre-coated with a specific capture antibody. After incubation at 37 °C, a Avidin-HRP-labeled detection antibody was added, followed by washing to remove unbound antibody. HRP-containing substrate solution was then added, allowing for a color change catalyzed by HRP. The reaction was stopped with an acidic termination solution, and absorbance was measured at 450 nm. IL-6, IL-1β and CCL-2 concentrations were calculated from the standard curve. Alanine aminotransferase (ALT) Assay Kit (C009-2-1, Nanjing Jiancheng China) and Aspartate aminotransferase (AST) Assay Kit (C010-2-1, Nanjing Jiancheng China) following the Reitman-Frankel method. The assays detected pyruvate formation at 505 nm (ALT) or 510 nm (AST) after DNPH derivatization. Enzyme activities were calculated using pyruvate standards and expressed as U/L.

### Bioinformatics analysis

The potential protein targets of paeoniflorin, benzoylmesaconine, 6-shogaol, hypaconitine, atractylenolide, and benzoylaconine, were retrieved from three public databases: TCMSP https://old.tcmsp-e.com/tcmsp.php, Swiss Target Prediction http://swisstargetprediction.ch, and PubChem https://pubchem.ncbi.nlm.nih.gov. Oral bioavailability (OB), defined as the fraction of an orally administered drug that reaches systemic circulation, and drug-likeness (DL), which quantifies the structural similarity of a molecule to known drugs, were used as key pharmacokinetic screening criteria. To ensure pharmacological relevance, compounds with OB ≥ 30% and DL ≥ 0.18 were selected for further target identification. This filtering process facilitated the identification of biologically plausible protein targets associated with ZWT.

The disease targets associated with UC were retrieved from two public databases: OMIM https://omim.org/ and DisGeNET https://www.disgenet.org/. The search term “uremic cardiomyopathy” was used in both databases to identify relevant targets. The resulting target lists from OMIM and DisGeNET were combined, and duplicate entries were removed. The remaining non-redundant targets were considered as UC-related targets and used for subsequent analysis.

To investigate the biological functions of potential targets in UC, the DAVID (https://david.ncifcrf.gov/) database was utilized to perform GO analysis and KEGG pathway enrichment. GO analysis was conducted to examine biological processes (BP), cellular components (CC), and molecular functions (MF) associated with the identified targets. KEGG enrichment analysis was employed to identify key signaling pathways involved in the pathological mechanisms of UC. Subsequently, the GO and KEGG data were visualized using the Bioinformatics (http://www.bioinformatics.com.cn/) platform for comprehensive analysis.

### Cell cultures

Human renal proximal tubule cells (HK-2) were obtained from the Cell Bank of the Chinese Academy of Science (Shanghai, China) and cultured in F12/DMEM medium (KGL1201-500, KeyGEN BioTECH, China) containing 10% fetal bovine serum (30044333, Gibco,USA). When the cell density reached 80%, the cells were passaged into new culture dishes at a ratio of 1:2 to 1:3. To induce a kidney injury model, HK-2 cells were stimulated with 10 ng/mL TNFα (300-01A, Peprotech, USA) for 24 h.

Human acute monocytic leukemia THP-1 cells were abtained from the Suzhou Hycyte Biotechnology Co., Ltd and cultured in RPMI-1640 medium (KGL1501-500, KeyGEN BioTECH,China). When the cell density reached 4–10 × 10^5^ cells/mL, THP-1 cells were induced by 10 ng/mL PMA (ab120297, Abcama, UK) for 24 h differentiated into M1 macrophages.

Human cardiomyocytes (AC16) were obtained from the Cell Bank of the Chinese Academy of Science (Shanghai, China) and cultured in DMEM high-glucose medium (KGL1206-500, KeyGEN BioTECH, China). When the cell density reached 80%-90%, the cells were passaged into new culture dishes at a ratio of 1:2 to 1:4. All cells were cultured at 37 °C with 5% CO_2_. To establish a model of cardiac hypertrophy, AC16 cells were treated with 1 μM Angiotensin II (68,521–88-0, Sellcek, USA) for 48 h.

### Three-step sequential conditioned-medium co-culture assay

To investigate intercellular signaling among renal tubular epithelial cells, macrophages, and cardiomyocytes, a three-step sequential conditioned medium transfer model was established. HK-2 cells were stimulated with TNFα (10 ng/mL) in the presence or absence of benzoylaconine (20 μM), paeoniflorin (60 μM), or atractylenolide III (60 μM) for 6 h. After TNFα washout, the corresponding compounds were reapplied, and cells were incubated for an additional 12 h. The supernatant was then collected as HK-2 conditioned medium. THP-1 cells were differentiated into macrophages using PMA (10 ng/mL) for 24 h, then incubated with HK-2 conditioned medium for 24 h. Subsequently, macrophage-conditioned medium was collected and transferred to AC16 cardiomyocytes for 24 h prior to analyses.

### Molecular docking

Molecular docking simulations were performed using AutoDock Vina to investigate the binding affinity and orientation of the ligands within the protein active site. The crystal structures of the target proteins were obtained from the RCSB Protein Data Bank (PDB). Preparation of the receptor was carried out using AutoDockTools (ADT) 1.5.7, which included the removal of crystallographic water molecules, the addition of polar hydrogen atoms, and the assignment of Gasteiger partial charges. Atomic parameters were further defined by assigning AutoDock 4 (AD4) atom types. The grid box was centered on the coordinates of the co-crystallized ligand to define the binding pocket, ensuring the search space was focused on the biologically relevant site. Ligands were similarly prepared and converted to PDBQT format via ADT. For the docking process, the exhaustiveness was set to 8 to ensure an adequate global search of the conformational space, and the number of generated binding modes (num_modes) was set to 9. The binding poses were evaluated based on their predicted affinity (kcal/mol), and the conformation with the lowest binding energy was selected for subsequent interaction analysis.

### Statistical analysis

Data processing and graphing were performed using GraphPad Prism 8.0 software. Protein quantification performed using ImageJ software. Data are expressed as mean ± SD. Statistical analyses were conducted using an unpaired Student's t test for comparisons between two groups, and one-way analysis of variance (ANOVA) followed by Tukey's multiple comparisons test for comparisons among multiple groups. A *p*-value of < 0.05 was considered statistically significant.

## Supplementary Information

Supplementary Material 1.

## Data Availability

Data will be made available on request.

## Abbreviations

AC16: Human cardiomyocytes
CCL2: C–C motif chemokine ligand 2
CHF: Chronic heart failure
CKD: Chronic kidney disease
CO: Cardiac output
Cr: Creatinine
CRP: C-reactive protein
CXCL1: C-X-C motif chemokine ligand 1
EF: Ejection fraction
ESKD: End-stage kidney disease
FS: Fractional shortening
GCG: Glucagon
GO: Gene Ontology
HE: Hematoxylin–eosin
BUN: Blood urea nitrogen
HK-2: Human renal proximal tubule cells
IL-1β: Interleukin-1β
IL-6: Interleukin-6
LV: Left Ventricular
LVEDD: Left ventricular end-diastolic diameter
LVESV: Left ventricular end-systolic volume
LVPW: Left ventricular posterior wall
PCA: Principal component analysis
PEA: Proximity extension assay
PMA: Phorbol 12-myristate 13-acetate
TCM: Traditional Chinese Medicine
TGF-α: Transforming growth factor alpha
TNFα: Tumor necrosis factor-α
UC: Uremic cardiomyopathy
HPLC: High performance liquid chromatography
UHPLC-Q/TOF–MS: Ultra-high performance liquid chromatography-quadrupole/time of fly-mass spectrometry
ZWT: Zhen-Wu-Tang
