# Supplementary material for: Zhen-Wu-Tang ameliorates uremic cardiomyopathy via targeting the kidney–heart inflammatory axis and suppressing CCL2/CCR2-mediated macrophage activation
Source: Chin Med. 2026 Mar 18;21:96. doi: 10.1186/s13020-026-01376-2 (PMC12998038; doi:10.1186/s13020-026-01376-2)
Supplement: Supplementary file 1 — Supplementary Material 1. [file 13020_2026_1376_MOESM1_ESM.docx]

**Zhen-Wu-Tang Ameliorates Uremic Cardiomyopathy via Targeting the Kidney-Heart Inflammatory Axis and Suppressing CCL2/CCR2-Mediated Macrophage Activation**

Yu Xu^a,1^, Jing Cai^b,1^, Yuan-Ming Fan^c^, Lian-Wen Qi^a,c*^, Lei Zhang^a,b*^

**Supplementary Figures S1-S7**

**Figure S1**

**
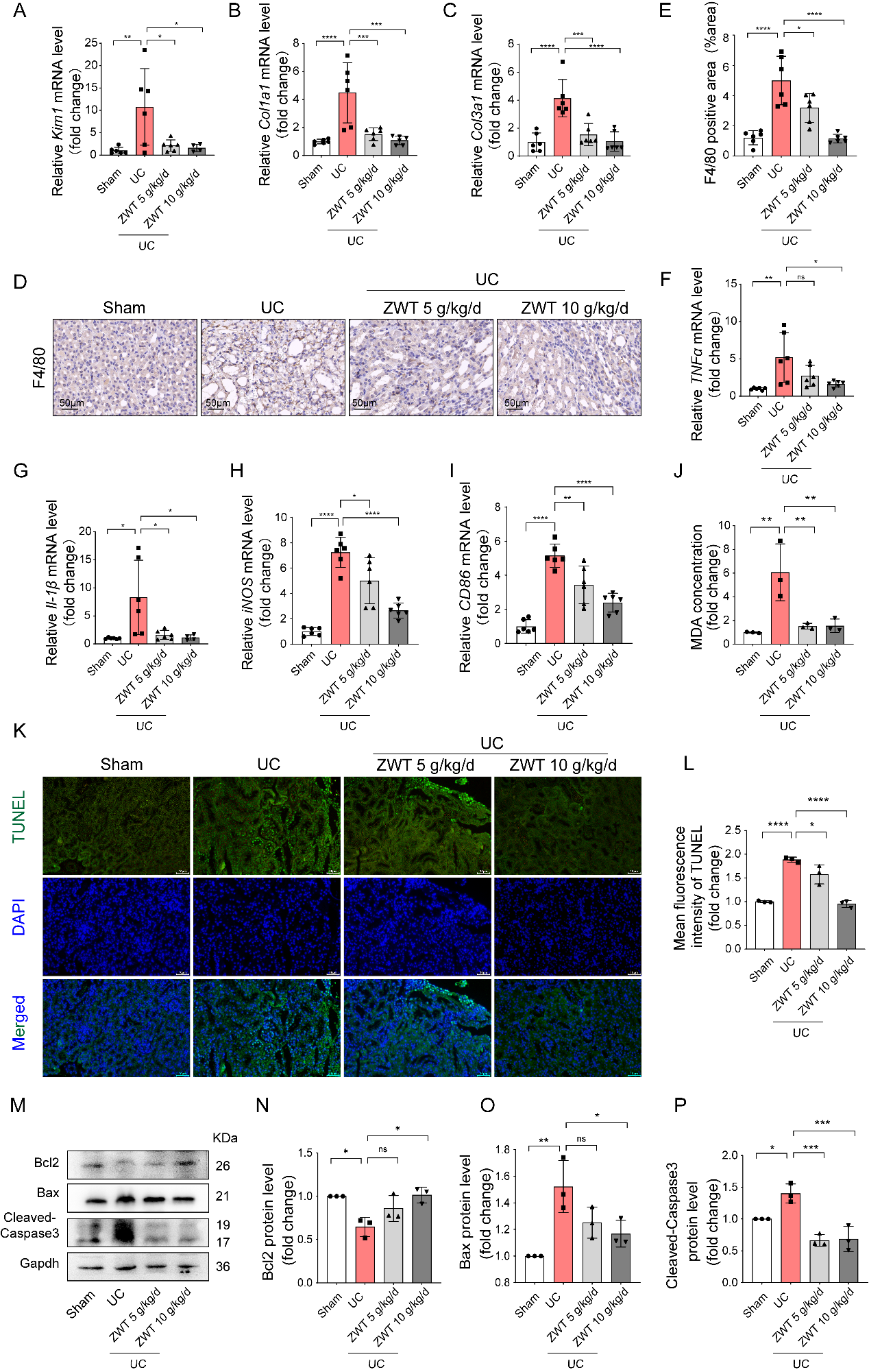
**

**Figure S1** **ZWT Attenuated Renal Injury and M1 Macrophage Polarization in UC Mice.** (**A-C**) Relative mRNA levels of kidney injury and fibrosis were assessed by qPCR, n = 6 samples per group. (**D**) F4/80 immunohistochemistry staining of kidney sections, n = 6 mouse kidneys per group, scale bar = 50 μm. (**E**) F4/80-positive area, n = 6 samples per group. (**F-I**) Relative mRNA levels of inflammation and M1 macrophage polarization-related markers in kidney tissues were measured by qPCR, n = 6 samples per group. (**J**) Malondialdehyde (MDA) concentration in renal tissues were measured by thiobarbituric acid colorimetric assay. n = 3 samples per group. (**K**) Representative TUNEL staining of kidney sections from each group. TUNEL-positive nuclei are shown in green, and nuclei were counterstained with DAPI (blue). Scale bar = 10 μm. n = 3 samples per group. (**L**) Quantification of TUNEL staining expressed as mean fluorescence intensity. (**M**) Representative western blot images analysis the expression of Bcl-2, Bax, and Cleaved-Caspase3 in renal tissues from the indicated groups. Gapdh served as an internal control. (**N-P**) Quantitative analysis of Bcl-2, Bax, and Cleaved-Caspase3 protein levels normalized to Gapdh. Protein quantification performed using ImageJ software. n = 3 samples per group. (**A-C**, **E-J**, **L**, and **N-P**) data were presented as mean ± SD, data were analyzed using one-way ANOVA with Tukey's test, with p ≤ 0.05 considered statistically significant. * *p* ≤ 0.05, ** *p* < 0.01, *** *p* < 0.001, **** *p* < 0.0001.

**Figure S2**


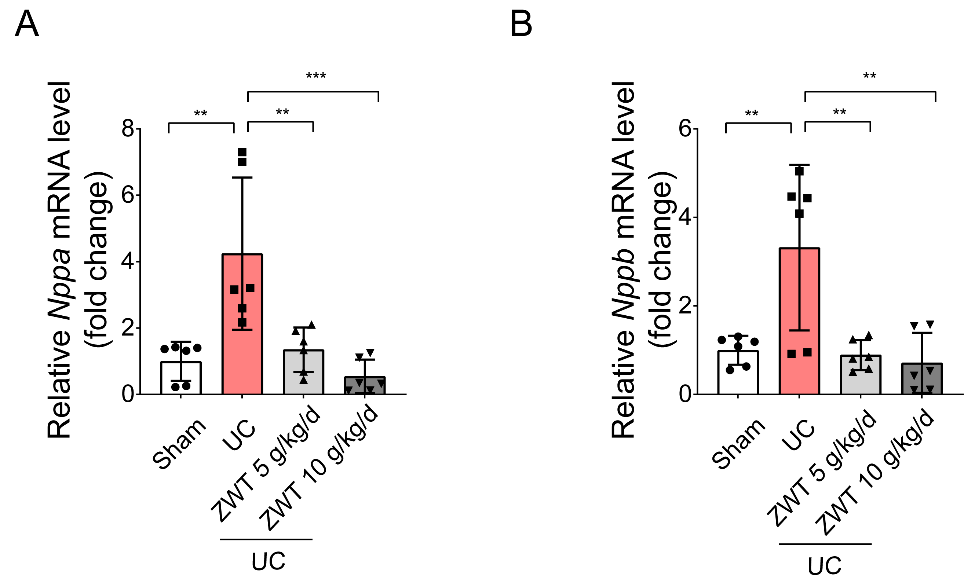


**Figure S2 ZWT Suppressed Cardiac Hypertrophy in UC Mice. (A-B)** Relative mRNA expression of *Nppa* and *Nppb* normalized to *18s* rRNA, n = 6 samples per group. All data were presented as mean ± SD, data were analyzed using one-way ANOVA with Tukey's test, with p ≤ 0.05 considered statistically significant. * *p* ≤ 0.05, ** *p* < 0.01, *** *p* < 0.001, **** *p* < 0.0001.

**Figure S3**


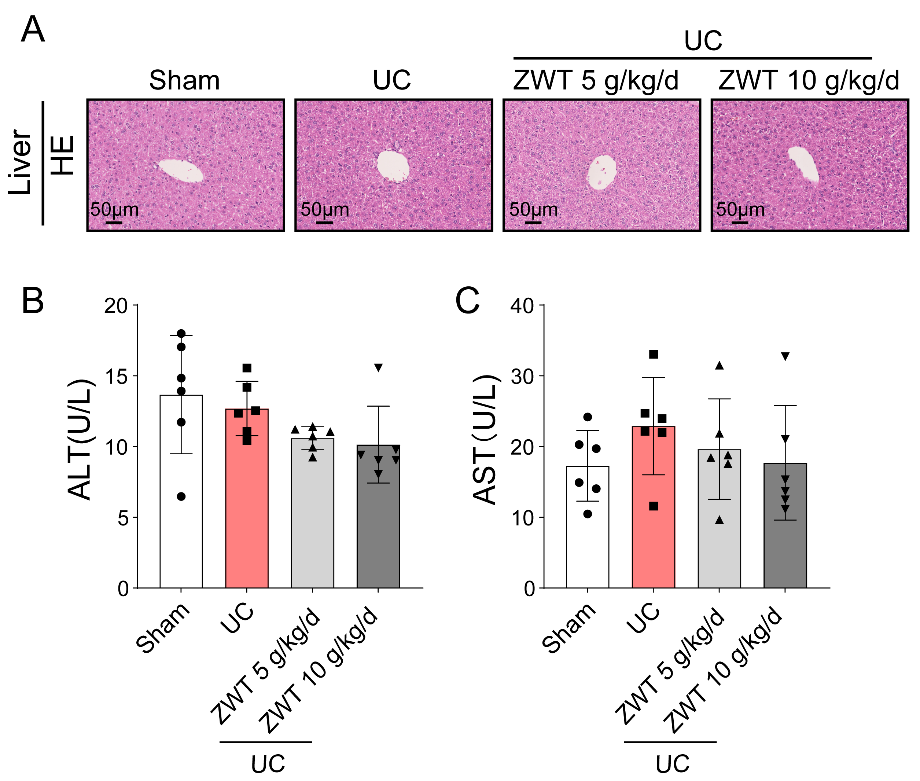


**Figure S3** **ZWT Exhibited a Favorable Safety Profile with No Signs of Hepatotoxicity.** (**A**) Representative liver sections stained with HE, n = 3 mouse livers per group, scale bar = 50 μm. (**B**) Alanine aminotransferase (ALT) levels and (**C**) Aspartate aminotransferasee (AST) levels in blood were measured by ELISA, n = 6 mice per group. All data were presented as mean ± SD, data were analyzed using one-way ANOVA with Tukey's test, with *p* ≤ 0.05 considered statistically significant. * *p* ≤ 0.05, ** *p* < 0.01, *** *p* < 0.001, **** *p* < 0.0001.

**Figure S4**

**
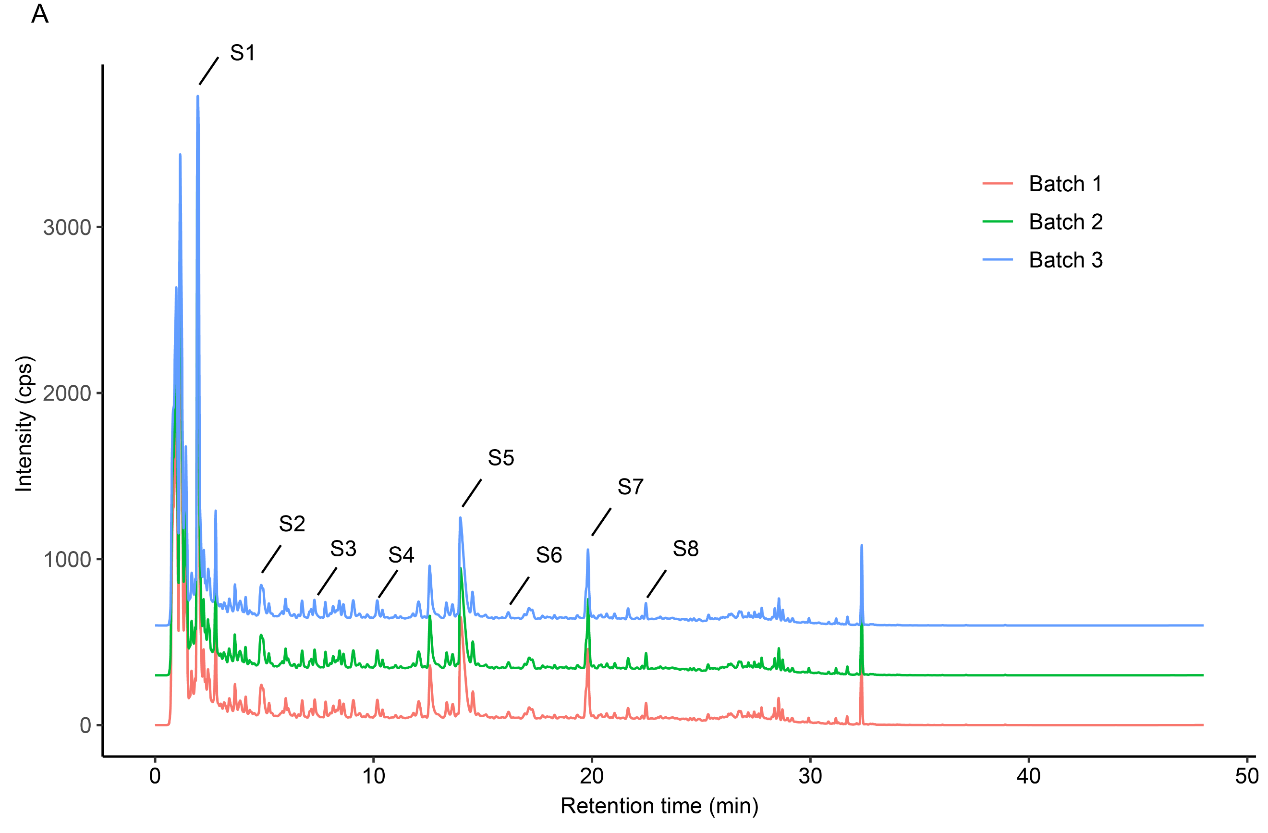
**

**Figure S4 HPLC Fingerprinting Analysis of ZWT. (A)** HPLC fingerprint of ZWT prepared in three independent batches.

**Figure S5**


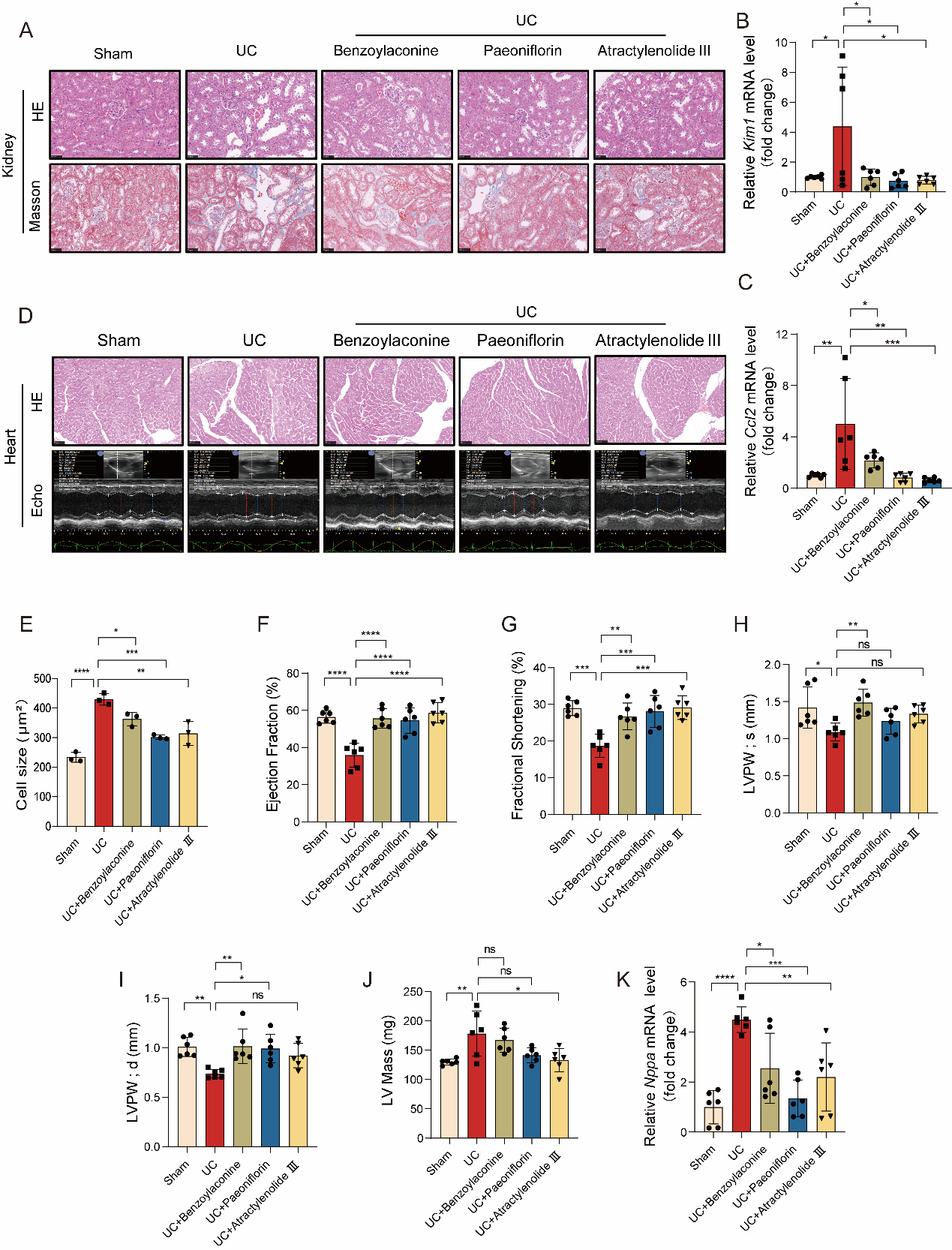


**Figure S5 Distinct Cardiorenal Protective Effects of Individual ZWT Monomers.** Mice were subjected to 5/6 nephrectomy and treated intragastrically for 8 weeks with benzoylaconine (10 mg/kg/d), paeoniflorin (50 mg/kg/d), or atractylenolide III (20 mg/kg/d). (**A**) Representative images of kidney sections stained with hematoxylin-eosin (HE) and Masson (scale bar = 50 μm; n = 3 mice per group). (**B, C**) Relative mRNA expression of *Kim1* and *Ccl2* in kidney tissue (n = 6 mice per group). (**D**) Representative images of HE-stained heart sections (n = 3 mice per group) and M-mode echocardiography (n = 6 mice per group). (**E**) Quantification of cardiomyocyte cross-sectional area from HE-stained sections (n = 3 mice per group). (**F–J**) Echocardiographic parameters: ejection fraction (EF), fractional shortening (FS), left ventricular posterior wall thickness at end‑systole (LVPW; s), end‑diastole (LVPW;d), and left ventricular mass (LV Mass) (n = 6 mice per group). (**K**) Relative mRNA expression of *Nppa* in heart tissue, normalized to 18s rRNA (n = 6 mice per group). Data were presented as mean ± SD and were analyzed by one‑way ANOVA with Tukey’s test. **p* < 0.05, ***p* < 0.01, ****p* < 0.001, *****p* < 0.0001.

**Figure S6**


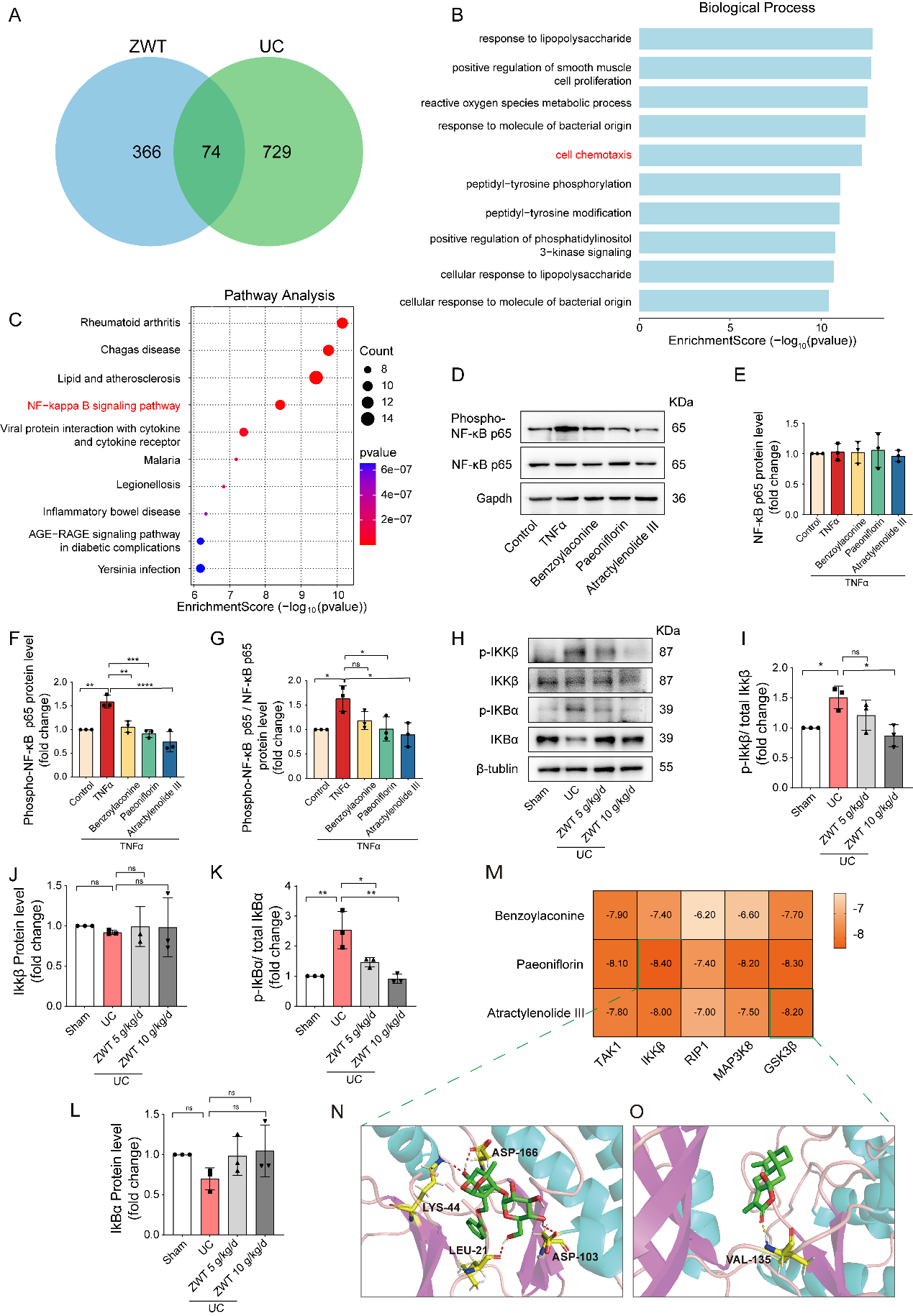


**Figure S6 ZWT inhibited NF-κB signaling activation.** (**A**) Venn diagram identifying the overlapping targets between ZWT and UC. (**B-C**) The top 10 Gene Ontology (GO) biological process (BP) terms (B) and the top 10 KEGG pathways (C) of the identified hub genes. (**D**) Western blot analysis of total and phosphorylated NF-κB p65 (p-p65) levels in HK-2 cells treated with TNF-α alone or in combination with benzoylaconine (20 μM), paeoniflorin (60 μM), or atractylenolide III (60 μM) for 24 h. Gapdh was used as the loading control. (**E–G**) Quantitative densitometric analysis of NF-κB p65 phosphorylation (n = 3). (**H**) Western blot analysis of total and phosphorylated IKKβ and IκBα levels in kidney tissues from 5/6 nephrectomy-induced UC mice treated with or without ZWT. (**I–L**) Quantitative densitometric analysis of IKKβ and IκBα phosphorylation and degradation (n = 3). (**M**) Heatmap showing binding scores (kcal/mol) of core ZWT components with key kinases, calculated via AutoDock Vina. Lower or more negative values indicate stronger binding affinities and higher structural stability. (**N-O**) Molecular docking models of Paeoniflorin with IKKβ (N) and Atractylenolide III with GSK3β (O). Ligands are represented as sticks within the protein binding pockets. Predicted hydrogen bonds and key interacting amino acid residues are indicated at the binding sites. Data in (**E-G** and **I-L**) were presented as mean ± SD and were analyzed by one-way ANOVA followed by Tukey's post hoc test. **p* < 0.05, ***p* < 0.01, ****p* < 0.001, *****p* < 0.0001.

**Figure S7**


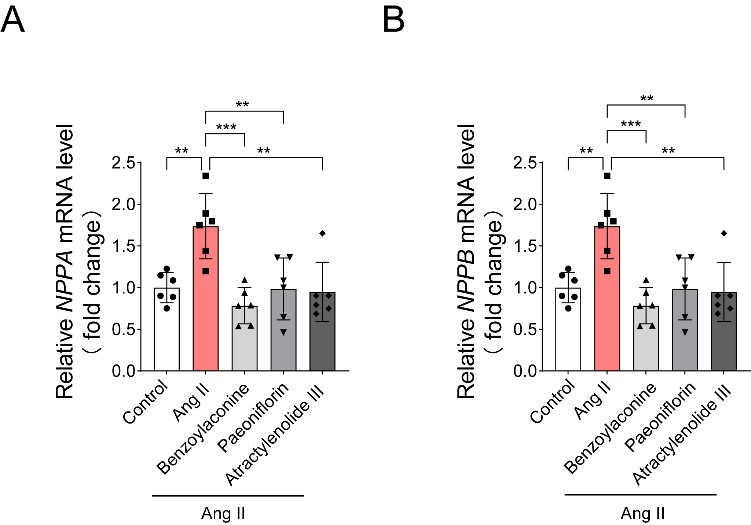


**Figure S7 Direct protective effects of ZWT active constituents on cardiomyocytes in vitro.** (**A-B**) To establish a cardiomyocyte hypertrophy model, AC16 cells were treated with Ang II (1 μM) for 48 hours with or without concurrent administration of three ZWT components: benzoylaconine (20 μM), paeoniflorin (60 μM), and atractylenolide III (60 μM). The mRNA expression of *NPPA* and *NPPB* was analyzed by RT-qPCR (n = 6 per group). All data were presented as mean ± SD, data were analyzed using one-way ANOVA, with p ≤ 0.05 considered statistically significant. * *p* ≤ 0.05, ** *p* < 0.01, *** *p* < 0.001, **** *p* < 0.0001.

**Supplementary Tables S1-S3**

**Table S1 Stability and repeatability of 8 characteristic peaks in ZWT.**

| **NO.** | **RT/min** | **RT-RSD** | **PA** | **PA-RSD** |
| --- | --- | --- | --- | --- |
| S1 | 1.947 | 0.18% | 25060.58 | 1.90% |
|  | 1.947 |  | 25461.23 |  |
|  | 1.953 |  | 26024.1 |  |
| S2 | 4.853 | 0.14% | 2018.39 | 0.44% |
|  | 4.86 |  | 2025.6 |  |
|  | 4.867 |  | 2007.86 |  |
| S3 | 7.293 | 0.10% | 633.52 | 0.81% |
|  | 7.3 |  | 643.73 |  |
|  | 7.307 |  | 639.93 |  |
| S4 | 10.167 | 0.03% | 760.91 | 1.15% |
|  | 10.173 |  | 744.3 |  |
|  | 10.173 |  | 756.77 |  |
| S5 | 13.973 | 0.07% | 8997.77 | 0.20% |
|  | 13.98 |  | 8961.85 |  |
|  | 13.993 |  | 8981.25 |  |
| S6 | 17.12 | 0.02% | 975.26 | 0.85% |
|  | 17.127 |  | 982.13 |  |
|  | 17.127 |  | 991.83 |  |
| S7 | 19.813 | 0.04% | 3380.11 | 0.27% |
|  | 19.813 |  | 3362.85 |  |
|  | 19.827 |  | 3375.94 |  |
| S8 | 22.467 | 0.02% | 438.72 | 1.07% |
|  | 22.473 |  | 429.63 |  |
|  | 22.473 |  | 432.55 |  |

Retention time (RT) and peak area (PA) of 8 characteristic peaks of ZWT, n = 3.

**Table S2 Chemical Characterization of 45 Major Components in ZWT**

| **No.** | **RT（min）** | **Formula** | **Predicted ions（*m/z*）** | **Measured ions（*m/z*）** | **Adduct** | **Major fragment ions（*m/z*）** | **Identification** |
| --- | --- | --- | --- | --- | --- | --- | --- |
| 1 | 0.907 | C10H13NO2 | 180.1021 | 180.1013 | M+H | 44.0481，65.0386，77.0385，79.0534，180.1010 | Salsolinol |
| 2 | 1.149 | C4H6O3 | 103.0393 | 103.0387 | M+H | 103.0390,43.0176 | Acetic anhydride |
| 3 | 1.337 | C6H13NO2 | 132.1021 | 132.1014 | M+H | 86.0961，69.0698，57.0573，44.0491，41.0381 | l-Isoleucine |
| 4 | 2.029 | C7H6O5 | 169.0135 | 169.0148 | M-H | 125.0244，123.0028，67.0187，95.0187 | Gallic acid |
| 5 | 2.296 | C6H6O3 | 125.0237 | 125.0248 | M-H | 124.0166，107.0138，97.0297，81.0340，79.0187，69.0339，53.0399，44.9981，51.0240，67.0188 | Pyrogallol |
| 6 | 6.104 | C23H37NO6 | 424.2689 | 424.2687 | M+H | 392.2414，374.2311，342.2060，324.1935， | Senbusine A |
| 7 | 8.185 | C15H14O6 | 291.0864 | 291.0815 | M+H | 139.0391，123.0347，67.0542，165.0534 | (+)-catechin* |
| 8 | 8.231 | C7H6O2 | 123.0444 | 123.0448 | M+H | 53.0367,43.0535,41.0382,95.0867,55.0530,67.0543 | Benzoic acid |
| 9 | 10.192 | C24H39NO7 | 454.2794 | 454.2795 | M+H | 436.2637，404.2422.418.2575 | Senbusine C* |
| 10 | 14.994 | C24H39NO6 | 438.2845 | 438.284 | M+H | 406.2566,392.2419,45.0329,374.2303,388.2467,420.2730 | Neoline |
| 11 | 16.298 | C25H41NO7 | 468.295 | 468.2941 | M+H | 418.2566,358.2305,436.2682 | Delsoline |
| 12 | 24.199 | C31H43NO10 | 590.2953 | 590.296 | M+H | 105.0331，75.0437，540.2578，508.2310，235.1108，196.1108，155.0848，138.0909，125.0226，108.0800，75.0437 | Benzoylmesaconine* |
| 13 | 26.093 | C32H45NO10 | 604.3109 | 604.3105 | M+H | 586.3006，572.2851，554.2748，522.2482，105.0336 | benzoylaconine* |
| 14 | 28.329 | C10H18O | 155.1431 | 155.1424 | M+H | 81.0692，69.0700，55.0532，41.0378，95.0855 | Geraniol |
| 15 | 29.05 | C30H32O12 | 585.1962 | 585.1924 | M+H | 319.1173，301.1063，213.0907 | Benzoylpaeoniflorin* |
| 16 | 29.46 | C34H47NO11 | 646.3214 | 646.3204 | M+H | 554.2709,586.3004,614.2920,91.0536,111.0801 | Aconitine* |
| 17 | 29.476 | C33H45NO10 | 616.3109 | 616.3109 | M+H | 584.2843，566.2725 | Hypaconitine* |
| 18 | 29.476 | C25H41NO9 | 500.2848 | 500.2848 | M+H | 616.3119,468.2620,450.2258,420.1772 | Aconine |
| 19 | 31.432 | C11H14O3 | 193.0861 | 193.0874 | M-H | 93.0325，41.0035，149.0603，107.0507 | Zingerone* |
| 20 | 32.191 | C17H26O4 | 295.1902 | 295.1901 | M+H | 277.1792,259.1692,153.1262,137.0595,41.0385,43.0539,55.0534,107.0481,151.0766； | 6-Gingerol* |
| 20 | 32.813 | C17H26O4 | 293.1746 | 293.1785 | M-H | 293.1785，293.1783，57.0353，147.0430 | 6-Gingerol* |

Continued Table 2

| **No.** | **RT（min）** | **Formula** | **Predicted ions（*m/z*）** | **Measured ions（*m/z*）** | **Adduct** | **Major fragment ions（*m/z*）** | **Identification** |
| --- | --- | --- | --- | --- | --- | --- | --- |
| 21 | 32.42 | C23H28O11 | 479.1545 | 479.1564 | M-H | 449.1404，327.1093，165.0812 | Albiflorin* |
| 22 | 32.42 | C23H28O11 | 479.1545 | 479.1564 | M-H | 327.1097，121.0287，77.0399 | paeoniflorin* |
| 23 | 32.844 | C31H43NO9 | 574.41 | 574.4149 | M+H | 542.2641,510.3324，524.1683，388.2423，105.0338 | Benzoylhypaconine* |
| 24 | 33.304 | C15H20O3 | 249.1485 | 249.1482 | M+H | 187.1116,173.0951,159.1161,147.0800,119.0853,121.1012,105.0695,93.0697,81.0693,79.0538,69.0699,67.0539,65.0387,41.0381 | atractylenolide Ⅲ* |
| 25 | 34.563 | C12H22O2 | 199.1692 | 199.1684 | M+H | 81.1570,111.1166,43.0537,67.0541,69.0700,81.0692,95.0826,41.0379 | 2-Lauroleic acid |
| 26 | 34.621 | C17H24O4 | 293.1746 | 293.1679 | M+H | 137.0595，275，1653，121.0631，151.0762，109.0646 | 6-Gingerdione |
| 27 | 34.639 | C15H22O4 | 265.1434 | 265.1446 | M-H | 57.0340，41.0008，55.0183，139.0740，111.0802，109.0672，93.0326，81.0346， | 4-Gingerol |
| 28 | 35.287 | C19H30O4 | 323.2214 | 323.2212 | M+H | 305.2106,137.0593,57.0695,67.0538,107.0847； | 8-Gingerol* |
| 28 | 32.266 | C19H30O4 | 321.2058 | 321.2072 | M-H | 303.1951，55.0181 | 8-Gingerol* |
| 29 | 35.777 | C20H30O4 | 335.2214 | 335.2211 | M+H | 67.0539,151.0747,317.2105 | Deoxyandrographolide |
| 30 | 35.91 | C15H20O2 | 233.1536 | 233.1535 | M+H | 187.1480,189.1629,215.1423,79.0538,157.1007,107.0856,105.0697,65.0383 | atractylenolide II* |
| 31 | 36.098 | C17H26O3 | 277.1797 | 277.1796 | M-H | 277.1801，41.0035，43.0183 | 1-(4-Hydroxy-3-methoxyphenyl)-3-decanone |
| 32 | 36.133 | C17H24O3 | 277.1797 | 277.1796 | M+H | 137.0597,122.0356,205.0872 | 6-Shogaol* |
| 33 | 36.626 | C33H52O5 | 529.2078 | 529.2087 | M+H | 511.3717,121.1016，405.1956，467.0078 | Pachymic acid* |
| 34 | 37.711 | C15H18O2 | 231.138 | 231.1377 | M+H | 79.0537，105.0695，229.1219，91.0536，65.0367，67.0537 | atractylenolide I* |
| 35 | 38.785 | C31H46O4 | 483.3462 | 483.3465 | M+H | 483.3463，419.3301，437.3428 | Poricoic Acid C |
| 36 | 38.904 | C31H46O4 | 483.3462 | 483.3465 | M+H | 465.3316，466.3378 | Polyporenic acid C* |
| 37 | 39.047 | C16H22O4 | 279.159 | 279.1586 | M+H | 205.0851，149.0226，41.0381，57.0697 | Diisobutyl phthalate |
| 38 | 39.047 | C16H22O4 | 279.159 | 279.1586 | M+H | 205.0851，41.0381，149.0704，205.0851，57.0697 | Dibutyl Phthalate |

Continued Table 2

| **No.** | **RT（min）** | **Formula** | **Predicted ions（*m/z*）** | **Measured ions（*m/z*）** | **Adduct** | **Major fragment ions（*m/z*）** | **Identification** |
| --- | --- | --- | --- | --- | --- | --- | --- |
| 39 | 40.205 | C21H24O5 | 355.1539 | 355.158 | M-H | 337.147 | Gingerenone A |
| 40 | 42.986 | C23H28O12 | 497.4234 | 497.4241 | M+H | 45.0342,480.3979,336.3263,283.1735 | Oxypaeoniflorin* |
| 41 | 44.066 | C13H22O2 | 211.1692 | 211.1691 | M+H | 121.1006，109.1007，95.0851，83.0848，81.0700，79.0533，69.0697，57.0339，55.0537 | Neryl propionate |
| 42 | 44.201 | C16H32O2 | 255.2316 | 255.233 | M-H | 237.2242，44.9981 | Palmitic Acid |
| 43 | 44.584 | C18H34O2 | 281.2472 | 281.2486 | M-H | 282.2552 | Oleic acid |
| 44 | 44.839 | C18H32O2 | 281.2472 | 281.2486 | M+H | 123.1130,81.0684 | Linoleic Acid |
| 45 | 45.639 | C20H40O2 | 313.3096 | 313.3147 | M+H | 295.3023 | Arachic acid |

*：The results were compared with the standard

**Table S3 34 Identification of Blood-Absorbed Active Components of ZWT**

| **No.** | **RT（min）** | **Formula** | **Predicted ions（*m/z*）** | **Measured ions（*m/z*）** | **Adduct** | **Major fragment ions（*m/z*）** | **Identification** |
| --- | --- | --- | --- | --- | --- | --- | --- |
| 1 | 0.985 | C9H11NO3 | 182.0812 | 182.0816 | M+H | 165.0546，136.0760，123.0442，119.0488，107.0493，95.0494，91.0545，77.0386，65.0385 | L-tyrosine |
| 2 | 1.018 | C6H13NO2 | 132.1019 | 132.1021 | M+H | 86.0968，69.0697，57.0575，44.0495，41.0388 | L-Ile |
| 3 | 1.322 | C6H6O3 | 125.0244 | 125.0227 | M-H | 81.0316，97.0248，69.0336 | Pyrogallol |
| 4 | 1.341 | C9H11NO2 | 166.0863 | 166.0859 | M+H | 149.0572,120.0802,121.0837,103.0514,93.0696,79.0543 | L-Phenylalanine |
| 5 | 1.401 | C7H6O5 | 171.0288 | 171.0262 | M+H | 129.0172，99.0033 | Gallic acid |
| 6 | 2.494 | C9H11NO2 | 164.0717 | 164.07 | M-H | 147.0433，103.0540，91.0512，72.0079，61.9858，41.9915 | Phenylalanine |
| 7 | 7.807 | C6H12O6 | 179.0561 | 179.0525 | M-H | 59.0131，71.0123，87.0077，89.0243 | Polymannose |
| 8 | 7.922 | C6H12O6 | 179.0561 | 179.0516 | M-H | 113.0216，71.0134，89.0241，59.0124 | Methose |
| 9 | 14.331 | C23H28O11 | 479.1559 | 479.1506 | M-H | 327.1072,121.0293,165.0534,77.0373 | paeoniflorin |
| 10 | 18.647 | C7H6O3 | 137.0244 | 137.0235 | M-H | 93.0342，65.0390 | Salicylic acid |
| 11 | 24.293 | C31H43NO10 | 590.296 | 590.292 | M+H | 572.2827,540.2560,105.0344 | Benzoylmesaconine |
| 12 | 28.544 | C17H24O3 | 277.1798 | 277.1798 | M+H | 137.0600，122.0363，146.0714，151.0750，138.0677 | 6-Shogaol |
| 13 | 28.657 | C21H32O4 | 349.2373 | 349.2365 | M+H | 331.2239,313.2167,121.0652,109.0639 | 10-Gingerdione |
| 14 | 28.735 | C11H14O3 | 195.1016 | 195.1019 | M+H | 177.0942，149.0953，121.0649，111.0417，91.0543 | Zingerone |

Continued Table 3

| **No.** | **RT（min）** | **Formula** | **Predicted ions（*m/z*）** | **Measured ions（*m/z*）** | **Adduct** | **Major fragment ions（*m/z*）** | **Identification** |
| --- | --- | --- | --- | --- | --- | --- | --- |
| 15 | 29.304 | C15H22O4 | 267.1591 | 267.1591 | M+H | 207.1018,221.1171,107.0486,79.0544 | 4-Gingerol |
| 16 | 29.398 | C33H45NO10 | 616.3116 | 616.311 | M+H | 556.2902，524.2625，557.2939，338.1753 | Hypaconitine |
| 17 | 30.868 | C15H18O2 | 231.138 | 231.1378 | M+H | 213.1265，203.1444，185.1334，121.1027，123.0439，105.0697，107.0873，109.1017 | atractylenolide Ⅰ |
| 18 | 31.371 | C15H20O2 | 233.1536 | 233.1536 | M+H | 215.1426，187.1480，177.0913，159.1163，157.1040，125.0597，107.0852，79.0545，67.0540，65.0379 | atractylenolide II |
| 19 | 31.754 | C24H36O5 | 405.2636 | 405.263 | M-H | 387.2521,369.2413,285.1841,201.1650 | Lovastatin |
| 20 | 31.894 | C17H24O4 | 293.1747 | 293.1744 | M+H | 275.1642，137.0605，177.0913，179.0731 | 6-Gingerdione |
| 21 | 32.21 | C30H40O2 | 433.3101 | 433.3131 | M+H | 415.2914，379.2959 | Citraurin beta |
| 22 | 32.343 | C11H22O | 171.1741 | 171.1738 | M+H | 97.1043，83.0850，67.0535 | Mnk |
| 23 | 34.541 | C15H20O3 | 249.1485 | 249.1476 | M+H | 119.0871，121.1027，105.0706，93.0701，91.0544，81.0701，79.0555，69.0705，41.0386 | atractylenolide Ⅲ |
| 24 | 35.615 | C10H12O | 149.0961 | 149.0956 | M+H | 115.0560,121.0654,105.0690,93.0697,91.0542,77.0386,79.0543 | Terragon |
| 25 | 35.728 | C20H30O4 | 333.2071 | 333.2043 | M-H | 315.1917，289.2152 | Deoxyandrographolide |
| 26 | 36.978 | C32H45NO10 | 604.3116 | 604.3141 | M+H | 586.305,522.2457 | benzoylaconine |
| 27 | 37.983 | C18H32O2 | 281.2475 | 281.2481 | M+H | 221.2243,179.1755,149.1329,135.1181,125.1313 | Linoleic acid |
| 28 | 40.752 | C12H22O2 | 199.1693 | 199.1693 | M+H | 111.1169,95.0854,83.0850,69.0700 | [(3S)-3,7-dimethyloct-7-enyl] acetate |
| 29 | 40.758 | C12H22O2 | 199.1693 | 199.1699 | M+H | 111.1164，109.1027，93.0694，83.0850，67.0535 | 2-Lauroleic acid |

Continued Table 3

| **No.** | **RT（min）** | **Formula** | **Predicted ions（*m/z*）** | **Measured ions（*m/z*）** | **Adduct** | **Major fragment ions（*m/z*）** | **Identification** |
| --- | --- | --- | --- | --- | --- | --- | --- |
| 30 | 41.289 | C20H30O2 | 303.2319 | 303.2315 | M+H | 189.1634，267.2114，285.2206，109.0638，175.1156，139.1112 | Methyltestosterone |
| 31 | 41.827 | C14H26O2 | 227.2006 | 227.2022 | M+H | 109.1025，95.0870，97.1015 | [(3R)-3,7-dimethyloct-6-enyl] butanoate |
| 32 | 42.888 | C20H36O2 | 309.2788 | 309.2765 | M+H | 273.2534,163.1449,137.1300,123.1183 | 11,14-eicosadienoic acid |
| 33 | 44.148 | C16H22O4 | 279.1591 | 279.1596 | M+H | 205.0883，149.0236，121.0267，93.0328 | Diisobutyl phthalate |
| 34 | 44.411 | C17H26O4 | 295.1904 | 295.1963 | M+H | 55.0541，41.0386，43.0543，151.0754，109.0664，179.0689， | 6-Gingerol |
